# Self-Fertilization, Inbreeding, and Yield in Alfalfa Seed Production

**DOI:** 10.3389/fpls.2021.700708

**Published:** 2021-07-06

**Authors:** Molly E. Dieterich Mabin, Johanne Brunet, Heathcliffe Riday, Lauren Lehmann

**Affiliations:** ^1^Vegetable Crops Research Unit, United States Department of Agriculture-Agricultural Research Service, Madison, WI, United States; ^2^US Dairy Forage Research Center, United States Department of Agriculture-Agricultural Research Service, Madison, WI, United States

**Keywords:** alfalfa, floral display size, geitonogamous selfing, inbreeding depression, *Medicago sativa*, selfing rate, seed yield metrics

## Abstract

Selfing (self-pollination) is the ultimate form of inbreeding, or mating among close relatives. Selfing can create yield loss when inbreeding depression, defined as a lower survival and reproduction of inbred relative to outbred progeny, is present. To determine the impact of selfing in alfalfa (*Medicago sativa* L.), we quantified the selfing rate of 32 alfalfa seed production fields located in three regions, namely, the Pacific Northwest (PNW), the Central Valley of California (CEV), and the Imperial Valley of California (IMP). Selfing rates (the proportion of selfed seeds) varied between 5.3 and 30% with an average of 12.2% over the 32 seed production fields. In both the parents and their progeny, we observed an excess of heterozygotes relative to Hardy–Weinberg expectations. We detected notable levels of inbreeding in parents (0.231 ± 0.007 parental inbreeding coefficient) and progeny (0.229 ± 0.005). There were a 15% decrease in the number of seeds per stem (seed set) and a 13% decline in the number of seeds per pod in selfed relative to outcrossed stems, but negligible inbreeding depression for pods per raceme and seed weight. The number of racemes on selfed stems increased significantly in fields with greater selfing rates, supporting the presence of geitonogamous or among flower selfing. Despite the significant level of inbreeding depression, seed set did not decrease in fields with higher selfing rates, where the greater number of racemes on the selfed stems increased the seed set. The effects of the field selfing rate on the seed yield metrics were mostly indirect with direct effects of the number of racemes per stem. Available data indicate that the majority of selfing in alfalfa is pollinator-mediated, and thus, eliminating selfing in alfalfa seed production would require the selection of self-incompatible varieties, which, by eliminating inbreeding depression, would provide a 15% potential increase in seed yield and an increase in future hay yield.

## Introduction

Selfing or self-pollination, where ovules are pollinated by the plant’s own pollen, is the ultimate form of inbreeding or mating between relatives. Inbreeding can lead to inbreeding depression, where the survival and reproduction of offspring of related individuals are less than those of offspring of outcrossed individuals (Charlesworth and Willis, 2009). In diploids, selfing reduces heterozygosity by half each generation although the decline is slower in polyploids (Bartlett and Haldane, 1934; Dewey, 1966; Busbice, 1969). The increase in genetic homozygosity following inbreeding leads to the expression of recessive alleles with detrimental effects on plant traits and fitness (Charlesworth and Willis, 2009). Autotetraploid species are expected to have lower inbreeding depression relative to diploids, partly because of the lower rate of progression to homozygosity (Bever and Felber, 1992) and partly because they are better able to mask single-copy deleterious mutations (Otto and Whitton, 2000). However, studies on inbreeding depression of autotetraploid wild plants (Galloway et al., 2003; Galloway and Etterson, 2007) and crops (Jones and Bingham, 1995; Li and Brummer, 2009) often reveal a substantially higher-than-expected level of inbreeding depression, approaching the level found in diploid species (Busbice and Wilsie, 1966; Galloway and Etterson, 2007). This higher level of inbreeding depression cannot be explained solely by the expression of deleterious recessive alleles with increased inbreeding and has been partly ascribed to the loss of complementary gene interactions (Bingham et al., 1994; Jones and Bingham, 1995). Because selfing can lead to inbreeding depression and affect crop yield, it is important to examine the extent of selfing in seed production fields.

Alfalfa, *Medicago sativa* L., also known as lucerne, is grown for both seed and hay production in the United States. Alfalfa is valued at $9.3 billion, making it the third most valuable field crop in the United States (NAFA News, 2018). The demand for alfalfa is increasing with a growing global population and a higher demand for beef (Putnam, 2019). Therefore, an important goal for alfalfa producers is to increase hay and seed yields. However, hay yield increases have been slow in alfalfa – forage yield in the Midwest has barely changed over the last two decades (Wiersma, 2001; Brummer and Putnam, 2018). Changes in seed production have been mediated mostly *via* the acreages planted. For example, in the United States in 2017, 30 million kg of alfalfa seed was produced to meet the needs of alfalfa forage growers; this represents a 12% increase in alfalfa seed production compared to 2012 production (NASS, 2017). While various management practices have been identified to help maintain and improve hay and seed yields (Mueller, 2008; Brummer and Putnam, 2018), the potential impact of self-fertilization on alfalfa seed yield has received less attention.

Although alfalfa is commonly categorized as an outcrosser, most varieties are self-compatible, meaning that mature seeds are produced under self-fertilization. In alfalfa fields, selfing rate estimates are highly variable and may range from 9 to 47% (Johansen, 1963; Pedersen, 1968; Knapp and Teuber, 1993; Brown and Bingham, 1994; Riday et al., 2015). Selfing rate represents the proportion of seeds that result from self-pollination as opposed to outcross pollination (pollen from a distinct plant). However, some of this variation could result from the different methods used to estimate selfing, including flower color polymorphisms (Burkart, 1937; Johansen, 1963; Bradner and Frakes, 1964; Pedersen, 1968), allozymes (Knapp and Teuber, 1993), and microsatellite markers (Riday et al., 2015). In addition, a variation in selfing rate could result from studies being done in different years and in different field plot types. To understand the extent of selfing and its potential impact, it is important to quantify selfing rate directly in alfalfa seed production fields, using a similar methodology, preferably using samples collected in the same year.

Selfing can lead to inbreeding depression, and severe inbreeding depression has been detected in alfalfa, as indicated by the following studies. Research that compared progeny phenotypes following an increasing number of selfed generations to their outcrossed parental plants observed a decrease in both seed and forage yields with increased selfing (Busbice, 1968; Melton, 1970; Gallais, 1984). For example, compared to the outcrossed parents, the number of seeds produced per plant, or per pod or flower (these categories are grouped here as “seed yield”), often decreased by 50% following one generation of selfing (Miller, 1966; Busbice, 1968; Gallais, 1984). Melton (1970) found an initial decrease of 42% in seed yield after one generation of selfing and a decrease of 71% after five generations. A similar trend has been observed for forage yield. The decrease in forage yield after one generation of selfing varied from 19 to 32% among studies (Kirk, 1927; Tysdal et al., 1942; Tysdal and Kiesselbach, 1944; Gallais, 1984). Kirk (1927) reported a 47% decrease in forage yield after three generations of selfing, and both Tysdal and Kiesselbach (1944) and Gallais (1984) noted approximately a 75% decrease after four generations of selfing, relative to the outcross parents. Given the potential negative impact of selfing on seed and hay yields, it is imperative to quantify the amount of selfing and inbreeding in alfalfa seed production fields.

In the current study, we estimated the selfing rate of 32 alfalfa seed production fields located in three major alfalfa seed-producing regions in the western United States. These regions include the Pacific Northwest (PNW), the Central Valley of California (CEV), and the Imperial Valley of California (IMP). Selfing rates were estimated during the same growing season and using a uniform methodology. We examined the level of genetic diversity within and among fields and among regions. We calculated the coefficient of inbreeding of maternal plants and progeny in each field. We compared various yield metrics, the number of seeds per stem (seed set), seeds per pod, pods per raceme, seed weight and racemes per stem, between selfed and outcrossed stems, and quantified inbreeding depression on the seed yield metrics. We examined the relationships between field selfing rate, number of racemes per plant, and the four seed yield metrics. This study provides the most comprehensive report of selfing rate in alfalfa seed production fields and quantifies its impact on the seed yield metrics. Identifying the mating system of a crop and its prevailing mode of selfing can guide the development of effective strategies to reduce selfing and increase yield.

## Materials and Methods

### Study Species, Field Sites, and Sample Collection

*Medicago sativa* L., also known as alfalfa or lucerne, is a perennial outcrossing plant. Most alfalfa cultivars are autotetraploid (2*n* = 4X = 32), and the species exhibits strong inbreeding depression (Li and Brummer, 2009). Bees are required for pollination and seed production (Bohart, 1957). Alfalfa flowers or florets are arranged in racemes, or clusters of flowers. When comparing alfalfa to many other plant species, a flowering stem can be equated to an inflorescence. In this case, racemes, the clusters of flowers, can be equated to flowers on inflorescences, and each flower within a raceme may be referred to as a floret. In botanical terms, a floret is a small flower that is part of a larger flower, a common feature of plant species in the family Asteraceae. Although a flower in alfalfa may not be a true floret, based on the botanical definition of the term, for the purpose of comparison with the structure of many flowering plants, the term “floret” represents a useful terminology. In this manuscript, the term “flower” refers to a floret, raceme to a cluster of florets, and flowering stem to an inflorescence. We use the number of racemes on a stem as a measure of floral display size, which typically describes the number of flowers on an inflorescence (Harder and Barrett, 1995; Karron et al., 2004).

Stems with leaves and mature fruits, called pods, were collected during the 2017 growing season from 32 alfalfa seed production fields located in three major alfalfa seed production areas, namely, the PNW (*N* = 11 fields), the CEV (*N* = 9 fields), and the IMP (*N* = 12 fields). Fifty individual stems were collected throughout each field, at distances large enough to prevent two stems from originating from the same plant, and thus, each stem represents a distinct plant in this study. In some of the PNW fields, only stems with mature pods (no leaves) were collected because these fields were desiccated prior to sample collection. Field-collected samples were shipped to Wisconsin where they were stored in a low-humidity room (20°C at 15–30% relative humidity) until processing.

### Sample Processing

Of the 50 stems collected per field, 40 were randomly selected for seed threshing. For fields in the CEV and IMP of California, for approximately 20 stems per field (range of 15–25), the number of racemes per stem, a measure of floral display size, was recorded prior to threshing. In addition, on five racemes per stem, the number of mature seed pods per raceme was counted and recorded. A pod is a fruit that developed from a flower (floret) on a raceme. These data could not be obtained for the PNW fields because only partial stems were collected in those fields. The 40 seed-bearing stems per field were individually threshed by hand using a wooden board and block with ridged plastic attached to them. Total seed weight (mg) was obtained for the seeds threshed from each stem, together with the weight of three independent sets of 10 mature seeds. The total number of seeds on a stem was calculated by dividing the total seed weight by the average weight of 10 seeds. We also calculated the average weight of a seed on a stem. The seeds from a stem were placed in an individual paper coin envelope labeled with the region, field number, and stem number, and envelopes were stored in a refrigerator until DNA processing. For samples from CEV and IMP, the number of seeds per stem (seed set), seeds per pod, pods per raceme, seed weight, and the number of racemes per stem were computed for each of the 20 stems per field.

### DNA Extraction and Microsatellite Loci

In each of the 32 fields, DNA was extracted from leaf tissue, or stem tissue, when leaves were not available, for 40 maternal parents and for a progeny array of eight seeds per mother for a total of 320 progeny per field. Over all fields, 1,274 extractions were performed from the maternal tissue and 10,190 extractions from seeds. Total DNA was extracted using modifications of the method of Štorchová et al. (2000). The methodology for the leaf or stem tissue is detailed in the study by Riday et al. (2013), and maternal tissue yielded 10–50 ng/μl of genomic DNA. For seeds, one seed and one 7/32'' chrome-steel bead were placed in each 1.0-ml well of a 96-well polypropylene block, and the wells were sealed with polypropylene strip caps. Tissue was powdered by grinding three times at 28 Hz for 2 min in a Mixer Mill MM400. The rest of the procedure was similar to the one detailed in the study by Riday et al. (2013). Seeds yielded 20–80 ng/μl of genomic DNA.

For each DNA sample extracted from the maternal tissue or seed, one multiplex PCR was performed with 14 primer pairs targeting 16 simple sequence repeat (SSR) loci. The 14 primer pairs with their accession name, fluorescent label, final primer concentrations (forward and reverse, μM), number of alleles found at each locus, and fragment sizes are listed in Table 1. The conditions for the PCR are further described in the study by Riday et al. (2015). Ten of the 14 primers and the remaining four forward primer sequences were obtained from the literature (Sato et al., 2005; Sledge et al., 2005; Li et al., 2011). The remaining four reverse primers (BE323955, AW16, BI28, and BG234; Table 1) were redesigned using the program Primer3^1^ to modify the size of the product so it could be identified following the multiplex PCR. Potential reverse primers suggested by Primer3 were tested to determine the best sequence to use for each reverse primer. Fragment sizes were determined on an ABI Prism 3730xl Genetic Analyzer as in the study by Riday et al. (2015), and the GeneMarker 1.91 software program (Soft-Genetics LLC, State College, PA, United States) was used to assign alleles at each microsatellite locus for each sample.

**Table 1:** Sixteen simple sequence repeat (SSR) loci used to genotype maternal alfalfa plats and progeny.

SSR primer pair	Accession name	Fluorescent label	Final concentration (μM)		Alleles	Fragment size range (bp)	Primer sequence
			Forward	Reverse			
BE323955	be323955	6-FAM	0.21	0.21	14	86–115	F: CACACTCTCTCTTCTCCGGTTC^1^ R: TTCGGGTCCGATGTAGTTTC^2^
AW690665^†^	aw690665	6-FAM	0.21	0.05	10	141–165	F: GGTTTTGGAGACATGACGGT^3^ R: GTGAAGACTTTGCGGTGGAT^3^
BI86	bi267671	6-FAM	0.21	0.21	15	206–236	F: GAAAAGAAATCACCCCGAAGAT^1^ R: CGTCGAAGTCAAAATCAATCTC^1^
AW170	aw695035	6-FAM	0.21	0.21	11	271–317	F: GATGCACTCACAGTGACAAACA^1^ R: TCAACGGTGTGAAGACGAAG^1^
AW16	aw685684	6-FAM	0.42	0.42	10	455–476	F: ATCGTCCCCACTGTGTCTTC^1^ R: GCAGCTTGACAAAGCATACG^2^
BE119^^^ BE119-B	be322616	HEX	0.21	0.21	9 13	139–155 162–183	F: GCTAGTTCTGCTCTCACTCTCATC^1^ R: CATTGTCTTTGTTGTGGAGGTG^1^
AW235	aw685316	HEX	0.21	0.21	8	187–202	F: CAGTTACGGTGTCATTCTCGTC^1^ R: TTGGGAGGAGTGTATGATGTTG^1^
BI131^†^	bi312375	HEX	0.21	0.05	10	242–266	F: GTTTTAGGAGAAGGAGGAGACG^1^ R: CAATAATCAACAACGGCAGAAG^1^
BI28	bi309553	HEX	0.21	0.21	23	360–413	F: TGAACCAACTGCACGAAGAG^1^ R: GCATAAGGGCTCCAAACAGA^2^
RCS5565	DE238450 *T. pratense*	TAMRA	0.21	0.21	4	95–103	F: ACAACCATGATGTGGGAATG^4^ R: AGATAGGAATTTGGGTCGGG^4^
BG280	bg647735	CAL Fluor Red 610	0.21	0.21	9	106–146	F: TCAGCAGTTAGTTTTGGTATGC^1^ R: TGTTGAAGTTGGAGTTTTGGTG^1^
BG222	bg585797	CAL Fluor Red 610	0.21	0.21	6	196–218	F: AGGGCTGATGAGGTGGATAAT^1^ R: ATCACGAGAACCGCCATAAGAT^1^
BI68	bi265211	CAL Fluor Red 610	0.42	0.42	4	245–260	F: CCATTCCAATCCACACTATCG^1^ R: ATCAGCGTAAATTCTGGCCTTA^1^
BG234-left^‡^ BG234-right	bg587084	CAL Fluor Red 610	0.42	0.42	13 9	356–386 417–438	F: CTGGAATACACCAAGCATGA^1^ R: TCATTGACTTCCCCAACCAT^2^

1 Primers in Sledge et al. (2005).

2 Reverse primers developed in the current study.

3 Primers in Li et al. (2011).

4 Primers in Sato et al. (2005).

† Asymmetric primer amounts added to obtain better amplification of all multiplex primers when combined.

^ BE119 primer pairs produced two separate amplification products designated BE119 and BE119-B.

‡ BG234 primer pairs produced two separate amplification products designated BG234-left and BG234-right.

We determined the total number of alleles at each microsatellite locus and calculated the polymorphic information content (PIC) of each locus in the parents and in the progeny using the software program POLYGENE (Huang et al., 2020). A PIC value is calculated based on the number and relative frequency of alleles and ranges from 0 to 1; a value of 0 indicates a monomorphic locus, and a value of 1 indicates a locus with many alleles at equal frequencies (Smith et al., 1997).

### Selfing Rate

We estimated the selfing rate for each of the 32 fields for this tetraploid species using a program developed by Riday, presented in the study by Riday et al. (2013). This program determines whether each seed resulted from a selfed or an outcrossed event. A seed is determined to be the result of self-fertilization if all progeny alleles are observed in the maternal genotype at all microsatellite loci. If an allele not present in the mother is observed in the progeny at any of the microsatellite loci, the seed is considered outcrossed. While it is possible for an individual to be falsely identified as selfed, which is most common with high-frequency alleles as they are likely to be present in both the mother and the father, this probability decreases dramatically as the number of microsatellite markers increases, such that for 16 loci the probability of a false identification as selfed is 1.10 E-5 (Riday et al., 2013). A field selfing rate in this study represents the proportion of self-fertilized seeds in the 320 seeds genotyped per field. We estimated the selfing rate of each field and compared the mean field selfing rate among the three seed-producing regions (PNW, CEV, and IMP) using a one-way ANOVA. We calculated individual selfing rates, based on the eight seeds genotyped per plant, and examined whether the proportion of selfed plants in a field and the proportion of selfed seeds per plant increased with greater field selfing rate using linear regressions (lm function in R). Analyses were done using R version 4.0 (R Core Team, 2020).

### Genetic Diversity

To measure the level of genetic diversity in a field, we calculated the number of alleles per locus and the level of heterozygosity. To adjust for differences in sample sizes between parents and progeny, the number of alleles for the progeny in each field was corrected for the sample size of the parents (generally *N* = 40; Supplementary Table 1) using the rarefaction method of Hurlbert (1971), as described in the study by Petit et al. (1998). Expected heterozygosity (H_E_) and observed heterozygosity (H_O_) were calculated for each field with the software program POLYGENE v1.0b (Huang et al., 2020), assuming random chromosome segregation as double reduction is rare in alfalfa (Julier et al., 2003). The POLYGENE software program facilitates genetic analysis of autopolyploids using allelic phenotypes (i.e., microsatellites without known allele dosage; Huang et al., 2020). Expected heterozygosity, also called gene diversity, describes the proportion of heterozygotes expected under Hardy–Weinberg equilibrium.

To examine how the genetic variation was distributed among individuals within fields, among fields within regions and among regions, we performed an analysis of molecular variance (AMOVA; Excoffier et al., 1992). The AMOVA was calculated based on the maternal genotypes, assuming a stepwise mutation model (SMM) distance for microsatellites (Slatkin, 1995) and using the weight genotype method in the software package POLYGENE (Huang et al., 2020). We compared the level of genetic diversity among the three geographic regions by contrasting the number of alleles per locus and H_O_ per field among the three regions using ANOVAs and *post hoc* Tukey’s multiple means comparison. A separate manuscript will examine the population genetic structure of these 32 seed production fields and contrast the genetic diversity and inbreeding coefficients between the dormant and nondormant alfalfa varieties.

The average number of alleles per locus per field between parents and progeny was compared using a paired *t*-test. To determine whether there was an excess of homozygotes in the progeny, as expected with selfing, we contrasted H_O_ to H_E_ for the progeny over each field using paired *t*-tests. We also compared H_O_ to H_E_ in the parents to determine the pre-mating condition. Finally, to establish whether H_O_ increased with field selfing rate, we performed linear regressions of observed level of heterozygosity of progeny against field selfing rate using the lm function in R. All analyses were done using R version 4.0 (R Core Team, 2020).

### Coefficient of Inbreeding

The coefficient of inbreeding quantifies the probability that alleles at a given locus are identical by descent (i.e., come from the same ancestor). For each field, the coefficient of inbreeding (F_Z_) was calculated for both parents and progeny using the polyploid-corrected multilocus estimator of Hardy (2016). This method estimates the inbreeding coefficient from marker genotypes and is robust to cases where the exact allele dosage cannot be determined, as is typically the case for microsatellite data in tetraploid organisms. We contrasted F_Z_ among the alfalfa seed production regions using one-way ANOVAs and did this for the parental and progeny generations. In addition, to determine if F_Z_ was greater in the progeny relative to the parents, as would be expected with selfing, we compared F_Z_ between the parental and progeny generations for each field using paired *t*-tests. Finally, because higher selfing can increase inbreeding, we examined the relationship between field selfing rate and progeny F_Z_ using a linear regression (lm function in R). Analyses were done with R version 4.0 (R Core Team, 2020).

### Yield Metrics

We contrasted each of the five yield metrics, seed set, number of seeds per pod, number of pods per raceme, seed weight and number of racemes per stem, between regions, among fields within region and between selfed and outcrossed plants (mating system) using a three-way ANOVA with proc GLM in SAS version 9.4 (SAS Institute, 2016). The two regions were CEV and IMP of California, with nine and 12 fields sampled per region, respectively. Selfed plants were those that had any selfed progeny, while outcrossed plants had no selfed progeny, when we examined the eight seeds genotyped per plant. We used plants for which both a genotype and yield metrics data were available in these analyses (*N* = 403 stems) and 211 stems were selfed (5–18 stems per field) and 192 were outcrossed (1–15 stems per field) (Supplementary Data). For each of the yield metrics, except for the number of racemes per plant, we quantified inbreeding depression (*δ*) as *δ* = 1‐ (w_s_/w_o_), where w_s_ is the mean fitness of the selfed and w_o_ of the outcrossed individuals, here expressed as 1‐ (mean value of the trait for selfed/mean value of the trait for outcrossed stems; Johnston and Schoen, 1994). Because the number of racemes is measured prior to selfing or outcrossing of the parents, the values between selfed and outcrossed stems could not be used as a measure of inbreeding depression for this yield metric. Based on the high inbreeding depression previously reported in alfalfa, we predicted higher values for seed set, seeds per pod, pods per raceme, and seed weight for outcrossed relative to selfed plants.

We examined the relationships between field selfing rate and each yield metric using the regression analysis with proc GLM in SAS version 9.4 (SAS Institute, 2016). For each yield metric, we first examined the regression over both regions before determining the relationships within each region. We were also interested in whether the pattern between field selfing rate and a yield metric differed between selfed and outcrossed stems, and thus, we performed separate regressions for the selfed and outcrossed stems in these fields first over both regions and then within each region. Because we obtained statistically significant regressions between field selfing rate and the number of racemes per stem, and between the number of racemes per stem and the four seed yield metrics, we also performed multiple regressions with the number of racemes and field selfing rate as independent factors and each of the four remaining yield metrics as dependent factors. A multiple regression will determine the impact of field selfing rate when the number of racemes is held constant, and vice versa. The stems in these analyses included all selfed and outcrossed stems (*N* = 403), with 192 outcrossed and 211 selfed stems.

## Results

### Microsatellites

Genotypes were obtained for 1,274 maternal parents and 10,190 seeds. A total of 168 alleles were identified across the 16 SSR loci with 138 alleles in the maternal parents and 165 alleles in the progeny. We detected 135 alleles in both the parents and the progeny, three alleles only occurred in the maternal parents, and 30 alleles were only detected in the progeny. The total number of alleles per locus varied among loci with an average of 8.6 ± 0.9 (mean ± SEM) alleles per locus in the parents (range 4–16) and 10.3 ± 1.2 alleles per locus in the progeny (range 3–23; Supplementary Table 1). After correcting for sample size differences between progeny and parents, overall, progeny had (mean ± SEM) 5.17 ± 0.05 alleles per locus per field relative to 5.05 ± 0.06 for the maternal parents (*t*_31_ = 2.92, *p* = 0.007). The PIC varied among loci but there was no difference in the mean PIC per locus between parents (0.603 ± 0.056) and progeny (0.607 ± 0.059; *t*_15_ = −0.75, *p* = 0.46), with a PIC range of 0.035–0.822 for the parents and 0.035–0.818 for the progeny.

### Selfing Rate

**Figure 1:**
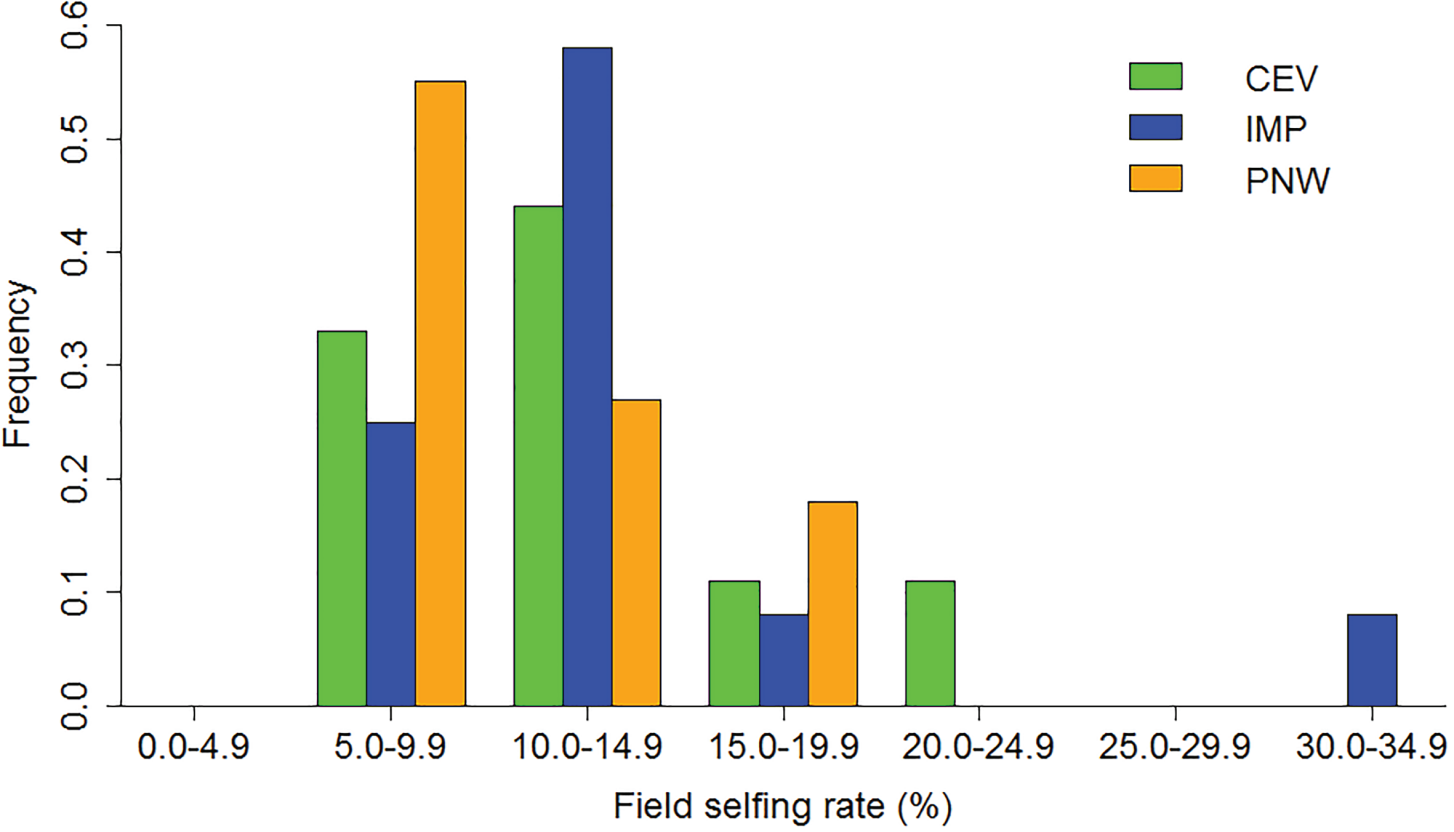
Frequency distribution of field selfing rates in three regions of alfalfa seed production. The regions are the Central Valley of California (CEV, *N* = 9 fields), the Imperial Valley of California (IMP, *N* = 12 fields), and the Pacific Northwest (PNW, *N* = 11 fields).

The mean field selfing rate was 12.2% with a range between 5.3 and 30.0% over the 32 alfalfa seed production fields (Figure 1; Supplementary Table 2). The mean field selfing rate did not differ among alfalfa seed production regions (PNW, CEV, and IMP; *F*_2,29_ = 1.34, *p* = 0.28; Table 2). The range in selfing rate was widest (8.1–30.0%) in the IMP of California and narrowest (5.3–16.9%) in the PNW (Figure 1). Both the proportion of mothers producing selfed seeds (*Y* = 2.53*x* + 22.35, *R*^2^ = 0.81, *F*_1,30_ = 132.9, *p* < 0.001) and the proportion of selfed seeds in selfed mothers (*Y* = 0.006*x* + 0.15, *R*^2^ = 0.563; *F*_1,30_ = 40.94, *p* < 0.001) increased with greater field selfing rate (Supplementary Figure 1).

**Table 2:** Selfing rate and measures of genetic diversity in the three geographical regions.

Region	*N*	Selfing rate	Number of alleles		Observed heterozygosity (H_O_)		Coefficient of inbreeding (F_Z_)	
			Parents	Progeny	Parents	Progeny	Parents	Progeny
PNW	12	10.3^a^ ± 1.1	5.24^a^ ± 0.12	5.33^a^ ± 0.10	0.736^a^ ± 0.002	0.742^a^ ± 0.003	0.224^a^ ± 0.011	0.212^a^ ± 0.009
IMP	11	13.4^a^ ± 1.7	4.83^b^ ± 0.08	5.00^b^ ± 0.05	0.727^a^ ± 0.003	0.732^b^ ± 0.003	0.239^a^ ± 0.014	0.237^a^ ± 0.009
CEV	9	12.7^a^ ± 1.3	5.12^a,b^ ± 0.07	5.21^a,b^ ± 0.10	0.604^b^ ± 0.003	0.734^a,b^ ± 0.002	0.227^a^ ± 0.011	0.240^a^ ± 0.008
Overall	32	12.2 ± 0.9	5.05 ± 0.06	5.17 ± 0.05	0.695 ± 0.010	0.736 ± 0.002	0.231 ± 0.007	0.229 ± 0.005

Average field selfing rate (%) with standard error, average number of alleles per locus per field, average observed heterozygosity (H_O_), and coefficient of inbreeding (F_Z_) per locus per field, for the parents and progeny, for the three geographical regions. The PNW stands for the Pacific Northwest, IMP for the Imperial Valley, and CEV for the Central Valley of California. The variable *N* represents the number of fields sampled within each region. Different letters indicate statistically different regions based on Tukey’s multiple means tests.

### Genetic Diversity

The great majority of the genetic variation of the maternal parents occurred among plants within fields (92.30%), with only 0.04% of the variation among fields within a region and 7.66% among regions (Supplementary Table 3). The average number of alleles per locus per field differed among regions for both parents (*F*_2,29_ = 4.89, *p* = 0.015) and progeny (*F*_2,29_ = 4.23, *p* = 0.024; Table 2). In the maternal plants, there were more alleles per locus per field (mean ± SEM; 5.24 ± 0.12) in PNW than in IMP (4.83 ± 0.08; *p* = 0.01) but neither differed from CEV (5.12 ± 0.07; PNW – CEV *p* = 0.68, IMP – CEV *p* = 0.12; Table 2). Similar patterns were observed in the progeny (Table 2).

Observed heterozygosity (H_O_) also differed significantly among regions for both the parents (*F*_2,29_ = 607.5, *p* < 0.001) and the progeny (*F*_2,29_ = 3.90, *p* = 0.032; Table 2). Observed heterozygosity in the parents was significantly less in CEV relative to PNW (CEV – PNW, *p* < 0.001) and IMP (CEV – IMP, *p* < 0.001) but did not differ between PNW and IMP (*p* = 0.09; Table 2). For the progeny, H_O_ was largest in PNW and significantly different from IMP (*p* = 0.04) but not from CEV (*p* = 0.10), while progeny H_O_ did not differ between IMP and CEV (*p* = 0.96; Table 2).

The level of observed heterozygosity per field (mean ± SEM; 0.695 ± 0.010) was greater than the level of expected heterozygosity (0.623 ± 0.004) by an average of 0.072 for the parents (*t*_31_ = 11.28, *p* < 0.001) and 0.092 for the progeny (*t*_31_ = 117.67, *p* < 0.001; 0.736 ± 0.002 for H_O_ and 0.644 ± 0.001 for H_E_; Table 2). Greater H_O_ relative to H_E_ indicates an excess of heterozygotes relative to Hardy–Weinberg equilibrium expectations.

### Coefficient of Inbreeding

The average coefficient of inbreeding per field did not vary among regions for the parents (*F*_2,29_ = 0.45, *p* = 0.64) or the progeny (*F*_2,29_ = 3.10, *p* = 0.06; Table 2). The parental inbreeding coefficient with a mean of 0.231 ± 0.007 and a range between 0.147 and 0.283 over all fields did not differ statistically from the progeny inbreeding coefficient with a mean of 0.229 ± 0.005 and a range from 0.154 to 0.292 (*t*_31_ = 0.24, *p* = 0.81; Supplementary Table 2). The progeny inbreeding coefficient increased with field selfing rate when the field with the highest selfing rate (IMP_01, 30.0% selfing) was excluded (*Y* = 0.004*x* + 0.18, *R*^2^ = 0.21, *F*_1,29_ = 8.75, *p* = 0.006) but not when that field was included in the analyses (*Y* = 0.001*x* + 0.21, *R*^2^ = 0.005, *F*_1,30_ = 1.16, *p* = 0.29; Supplementary Figure 2).

### Yield Metrics

We observed a statistically significant difference between the two regions for all yield metrics except for average seed weight (Table 3). For seed set, seeds per pod, pods per raceme, and racemes per stem, fields in the CEV region had higher values relative to fields in the IMP region (Table 4). All of the five metrics for seed yield differed among fields (Table 3). Only seed set and the number of seeds per pod were statistically different between selfed and outcrossed stems (Table 3), and selfed stems had lower seed set and fewer seeds per pod relative to outcrossed stems (Table 4). Inbreeding depression was 0.15 for seed set and 0.13 for seeds per pod with negligible values for the other traits (Table 4).

**Table 3:** The impact of region (CEV and IMP), field within region, and mating system (selfed or outcrossed) on each seed yield metric, based on a three-way ANOVA.

Source	DF	Type III SS	Mean square	*F*	Pr > *F*
**Seed set**					
Region	1	8423325.623	8423325.623	99.29	**<0.0001**
Field (Region)	19	6207527.053	326711.950	3.85	**<0.0001**
Mating	1	407146.081	407146.081	4.80	**0.0291**
Region∗Mating	1	31548.130	31548.130	0.37	0.5424
Field∗Mating (Region)	19	1401471.707	73761.669	0.87	0.6217
**Pods per raceme**					
Region	1	30.0188339	30.0188339	6.55	**0.0109**
Field (Region)	19	244.4686271	12.8667698	2.81	**<0.0001**
Mating	1	5.5030776	5.5030776	1.20	0.2738
Region∗Mating	1	17.1652769	17.1652769	3.75	0.0537
Field∗Mating (Region)	19	60.0460604	3.1603190	0.69	0.8294
**Seeds per pod**					
Region	1	359.2845985	359.2845985	142.05	**<0.0001**
Field (Region)	19	245.4983043	12.9209634	5.11	**<0.0001**
Mating	1	20.7563265	20.7563265	8.21	**0.0044**
Region∗Mating	1	0.2672310	0.2672310	0.11	0.7453
Field∗Mating (Region)	19	34.5077017	1.8161948	0.72	0.8006
**Average Seed Weight (mg)**					
Region	1	0.07136429	0.07136429	0.44	0.5083
Field (Region)	19	10.92013492	0.57474394	3.53	**<0.0001**
Mating	1	0.00822829	0.00822829	0.05	0.8222
Region∗Mating	1	0.03729189	0.03729189	0.23	0.6325
Field∗Mating (Region)	19	1.97217324	0.10379859	0.64	0.8772
**Racemes per stem**					
Region	1	882.544421	882.544421	11.84	**0.0006**
Field (Region)	19	6995.179962	368.167366	4.94	**<0.0001**
Mating	1	56.774639	56.774639	0.76	0.3834
Region∗Mating	1	100.070804	100.070804	1.34	0.2473
Field∗Mating (Region)	19	1165.402218	61.336959	0.82	0.6795

The seed yield metrics include seed set or number of seeds per stem, pods per raceme, seeds per pod, average seed weight, and number of racemes per stem. The statistically significant values are in bold.

**Table 4:** Seed yield metrics per region (CEV and IMP) and per mating system (selfed and outcrossed).

	Region				Mating				
	CEV		IMP		Selfed		Outcrossed		*δ*
Yield metric	Mean	SE	Mean	SE	Mean	SE	Mean	SE	
Seed set	627.84	29.50	294.30^∗∗∗^	15.40	404.88	21.14	475.05^∗^	28.25	0.15
Seeds per pod	5.18	0.16	3.00^∗^	0.09	3.67	0.13	4.24^∗∗^	0.15	0.13
Pods	7.57	0.18	6.97^∗∗∗^	0.14	7.33	0.16	7.11	0.15	0.03
Seed weight	2.38	0.03	2.36	0.03	2.37	0.03	2.37	0.03	0.00
Raceme	17.90	0.79	14.55^∗∗∗^	0.57	15.85	0.64	16.16	0.71	NA

Means and SE for the five seed yield metrics for each region (CEV and IMP) and mating system (selfed and outcrossed) and associated inbreeding depression (*δ*). Inbreeding depression was measured as 1‐ (mean value selfed/mean value of outcrossed stems). Mating refers to whether a plant (stem) was selfed or outcrossed based on genotyping of eight seeds per stem. Inbreeding depression (*δ*) could not be measured for racemes per stem as this trait was measured on the parent prior to selfing or outcrossing. CEV stands for Central Valley, and IMP for Imperial Valley of California; NA means not available. The plant trait “seed set” is the number of seeds per stem; “pods” refers to the number of pods per raceme; “seed weight” is the average weight of a seed (mg); and “raceme” is the number of racemes per stem. Asterisks indicate statistically significant differences between regions or between selfed and outcrossed parents following the three-way ANOVA (Table 3).

∗ 0.01 ≤ *p* ≤ 0.05;

∗∗ 0.001 ≤ *p* < 0.01;

∗∗∗ *p* < 0.001.

**Figure 2:**
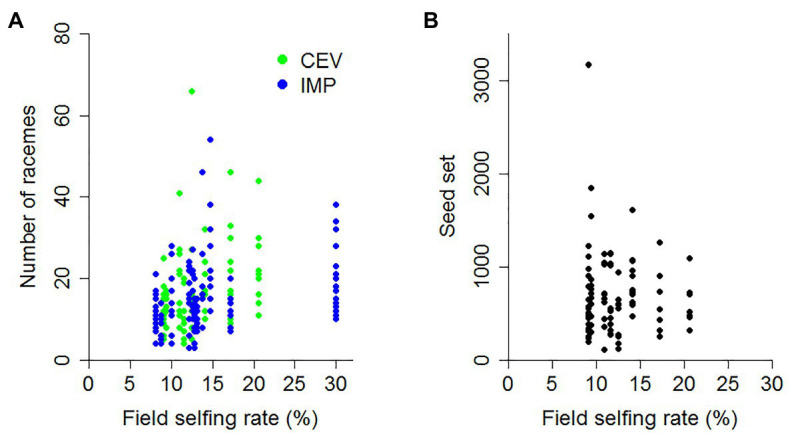
Field selfing rate and yield metrics. With an increase in field selfing rate, we observed **(A)** an increase in the number of racemes for selfed stems in CEV (*p* = 0.009, *r*^2^ = 8.2%) and IMP (*p* = 0.002; *r*^2^ = 7.3%), and **(B)** a decrease in the seed set for outcrossed stems in CEV (*p* = 0.03 and for the full model *r*^2^ = 67.1%, Table 5).

**Figure 3:**
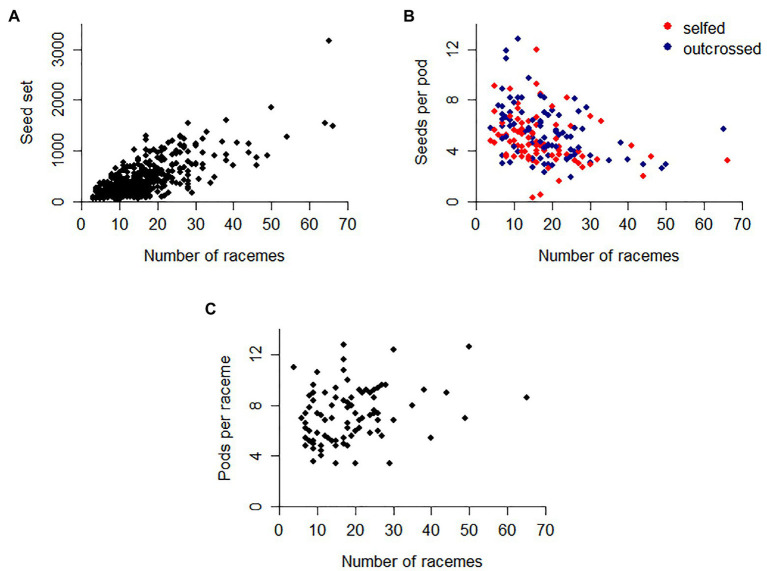
Number of racemes per stem and yield metrics. Stems with more racemes have **(A)** higher seed set (*p* < 0.0001), **(B)** fewer seeds per pod in CEV for both selfed (*p* = 0.01) and outcrossed (*p* = 0.0005) stems, and **(C)** more pods per raceme in CEV for outcrossed stems (*p* = 0.014). The *r*^2^ values for the full models are presented in Table 5.

We detected a statistically significant relationship between field selfing rate and the number of racemes per stem (Table 5). This relationship was present in both regions and, interestingly, was mostly driven by an increase in the number of racemes per stem on the selfed stems in the fields with higher selfing rates (Table 5; Figure 2A). Field selfing rate explained a greater proportion of the variance in the number of racemes for selfed relative to outcrossed stems (Table 5). For seed set, the number of racemes per stem explained 46% or more of the variance in seed set, while field selfing rate explained 4.3% or less (Table 5). When performing a multiple regression with field selfing rate and the number of racemes as independent factors, racemes significantly increased seed set in all cases (Table 5; Figure 3A), while field selfing rate negatively affected seed set for outcrossed stems in CEV (Table 5; Figure 2B). For the number of seeds per pod, in the multiple regression, field selfing rate only negatively impacted seeds per pod overall (Table 5, *p* = 0.042). In contrast, the number of racemes per stem negatively impacted seeds per pod for both outcrossed and selfed stems at CEV (Table 5, *p* = 0.0005 for outcrossed and *p* = 0.01 for selfed stems; Figure 3B). In single regressions, the number of racemes explained 13.8% of the variance in seeds per pod for outcrossed stems and 8.6% for selfed stems at CEV (Table 5). When examining the number of pods per raceme, the multiple regressions indicated that the number of racemes per stem had more impact relative to field selfing rate (Table 5). The number of racemes per stem increased the number of pods per raceme most strongly for outcrossed stems at CEV (Table 5; Figure 3C). For the average seed weight, none of the regressions were statistically significant (*p* > 0.19 in all cases). Overall, the number of racemes per stem had a greater impact on seed yield metrics relative to the field selfing rate.

**Table 5:** Field selfing rate, number of racemes, and seed yield metrics.

Dependent	Independent	Value	Overall	CEV	IMP	Out	Self	CEV out	CEV self	IMP out	IMP self
Raceme	Field SR (+)	**%**	**3.6**	**4.0**	**5.1**	**2.1**	**5.9**	1.9	**8.2**	1.9	**7.3**
		*p*	***	0.008	0.0005	0.04	0.0004	0.19	0.009	0.16	0.002
Seed set	Field SR (+)	**%**	0.28	0.15	**3.1**	0.5	0.8	0.05	2.5	3.7	**4.3**
		*p*	0.29	0.61	0.007	0.33	0.18	0.84 (−)	0.15	0.054	0.019
	Racemes (+)	**%**	**53.0**	**58.2**	**54.2**	**60.3**	**46.1**	**65.3**	**49.6**	**57.6**	**52.3**
		*p*	***	***	***	***	***	***	***	***	***
	Multiple	**%**	**53.7**	**59.5**	**54.3**	**60.5**	**46.7**	**67.1**	**49.8**	**58.4**	**52.3**
	Field SR (−)	*p*	**0.01**	**0.02**	0.83	0.35	0.15	**0.03**	0.57	0.18	0.845
	Racemes (+)	*p*	∗∗∗	∗∗∗	∗∗∗	∗∗∗	∗∗∗	∗∗∗	∗∗∗	∗∗∗	∗∗∗
Seeds/Pod	Field SR (−)	%	**1.5**	**2.3**	0.4	0.07	1.8	2.1	1.4	0.4	0.7
		*p*	0.015	0.048	0.33	0.71	0.046	0.17	0.28	0.56	0.37
	Racemes (−)	**%**	**1.4**	**10.6**	**2.4**	1.3	1.7	**13.8**	**8.6**	2.6	2.4
		*p*	0.018	***	0.02	0.12	0.059	0.0003	0.007	0.15	0.08
	Multiple	**%**	**2.4**	**11.3**	**2.5**	1.3	2.9	**14.7**	**8.8**	2.7	2.6
	Field SR (−)	*p*	**0.042**	0.23	0.64	0.89	0.11	0.33	0.74	0.43	0.65
	Racemes (−)	*p*	0.051	***	**0.03**	0.13	0.14	**0.0005**	**0.01**	0.13	0.12
Pods/Raceme	Field SR (+)	**%**	0.8	0.07	**2.3**	**2.2**	0.2	0.9	0.4	**3.9**	2.4
		*p*	0.07	0.74	0.021	0.04	0.44	0.38	0.58	0.049	0.08
	Racemes (+)	**%**	**1.9**	1.1	**1.7**	**4.6**	0.5	**7.2**	0.1	1.8	1.6
		*p*	0.006	0.17	0.047	0.003	0.3	0.01	0.77	0.18	0.15
	Multiple	**%**	**2.3**	1.1	**3.3**	**6.1**	0.6	**7.5**	0.4	5.1	3.2
	Field SR (+)	*p*	0.18	0.95	**0.05**	0.09	0.59	0.59	0.63	0.07	0.16
	Racemes (+)	*p*	**0.014**	0.19	0.13	**0.006**	0.38	**0.014**	0.88	0.27	0.31
Sample size		*N*	403	174	229	193	211	91	83	101	128

Single and multiple regressions on the different seed yield metrics. The value % indicates the percent of the variance in the dependent variable explained by the single and multiple regression models. The value of *p* is the probability statistics for a given regression model or for each factor in the multiple regression models (Multiple). Regressions were performed over both regions (Overall), for each region separately (CEV or IMP), for outcrossed (OUT) and selfed (SELF) stems overall, and for outcrossed and selfed stems within each region (e.g., CEV OUT). Regressions were performed using proc. GLM in SAS version 9.4 (SAS Institute, 2016). CEV is the Central Valley and IMP the Imperial Valley of California. The bolded % indicate the models that were statistically significant. For multiple regressions, significant factors are also bolded. The (+) or (−) next to an independent factor indicates the sign of the slope, i.e., an increase (+) or a decrease (−) in the dependent variable as the value of the independent factor increases. None of the regressions were statistically significant for average seed weight (all *p* > 0.19) and are thus not presented here. Sample size is the number of stems. ∗∗∗*p* < 0.0001; exact values of *p* are otherwise provided.

## Discussion

In plant species with a mixed mating system, both selfing and outcrossing are maintained in a population (Schemske and Lande, 1985; Holtsford and Ellstrand, 1989; Goodwillie et al., 2005; Brunet and Sweet, 2006; Whitehead et al., 2018). We obtained an average mean selfing rate of 12.2% over all seed production fields examined in this study. Using the average selfing rate over all alfalfa fields, and a definition of a mixed mating system as selfing rate between 20 and 80% (Goodwillie et al., 2005), classifies alfalfa as an outcrosser. However, Whitehead et al. (2018) warn against considering the mean selfing rate to ascribe a mating system to a plant species and stress the importance of considering the, often substantial, variation in selfing rate among populations. We observed considerable variation in selfing rate among fields, a range between 5.3 and 30% selfing, and this variation was detected within each of the three geographical regions. Most fields had 20% or less selfing and thus could be categorized as outcrossed. One field with a selfing rate of 30% would qualify as having a mixed mating system. Substantial variation in field selfing rates, between 9 and 53% selfing, has been detected in previous studies of alfalfa (Johansen, 1963; Pedersen, 1968; Knapp and Teuber, 1993; Brown and Bingham, 1994; Riday et al., 2015). However, in these studies, the level of selfing was estimated using varying methodologies, which can affect selfing rate estimates (Kehr, 1973; Steiner et al., 1992; Knapp and Teuber, 1993). Significant variation in individual plant selfing rates has also been reported in alfalfa, ranging from 0 to 52.2% with a mean of 11.8% in a previous study (Riday et al., 2015) and between 0 and 100% selfing with a mean of 11.8% over all individuals examined in this study, with the recognition that only eight seeds per individual were genotyped here. Variation in individual selfing rate also occurs in wild plant populations (Brunet and Eckert, 1998; Whitehead et al., 2018), and the variation in selfing rate among individuals and populations can help identify the factors that affect selfing rates (Brunet and Eckert, 1998; Brunet and Sweet, 2006; Whitehead et al., 2018). The current study represents the most extensive report on selfing rates in alfalfa seed production and indicates some level of selfing in each field examined. A previous study on two wild *Medicago* species in China, *M. lupulina* and *M. ruthenica*, reported the former as having a mixed mating system with a selfing rate of 29.5% and the latter as a selfer with a selfing rate of 95.8% (Yan et al., 2009). The species *M. truncatula* is highly selfed with close to 99% selfing (Siol et al., 2008). Future studies of selfing rate in a number of wild alfalfa populations (*M. sativa*) would determine whether wild populations are mainly outcrossed or have a mixed mating system. Such knowledge could help determine whether the domestication of alfalfa and its breeding process have influenced its mating system.

Selfing reduces the level of heterozygosity by half every generation in diploids although it takes 3.8 generations of selfing for a half reduction in heterozygosity in tetraploids such as alfalfa (Bartlett and Haldane, 1934; Dewey, 1966; Busbice, 1969). We expected the presence of selfing in alfalfa seed production fields to be associated with a deficiency of heterozygotes, at least in the progeny. We observed, however, a level of heterozygosity greater than predicted based on Hardy–Weinberg equilibrium and an excess of heterozygotes in both the parents and the progeny. Because the progeny were genotyped as seeds, the excess of heterozygotes suggests that some homozygous seeds do not reach maturity and thus the presence of early-acting inbreeding depression in the form of selection against homozygous seeds (Schemske et al., 1994). The excess of heterozygotes in the parents also suggests selection against homozygotes throughout the alfalfa life cycle from seed germination to flowering. Inbreeding depression is known to vary over the life cycle of plants (Husband and Schemske, 1996, 1997; Galloway et al., 2003; Galloway and Etterson, 2007). For example, early-acting inbreeding depression, i.e., on seed production, may be quickly reduced as a few large-effect recessive alleles are purged, while late-acting inbreeding depression, i.e., on survival, flowering, and biomass (i.e., hay production), may be due to many slightly detrimental recessive alleles and be more difficult to purge (Husband and Schemske, 1996). In autotetraploid alfalfa, the loss of complementary gene interactions is also likely to play a role in inbreeding depression (Bingham et al., 1994; Jones and Bingham, 1995). To fully understand the effect of inbreeding depression in a plant species, multiple traits must therefore be examined across its life cycle. In alfalfa, inbreeding depression has been shown to impact various stages of the life cycle, including seed production and hay yield (Aycock and Wilsie, 1968; Busbice, 1968; Melton, 1970; Gallais, 1984; Riday et al., 2017). In addition, selfing has also been shown to reduce the rates of flowering and winter survival (Riday et al., 2017), increase the number of days to bloom (Aycock and Wilsie, 1968; Posler, 1969), reduce plant height (Panella and Lorenzetti, 1966; Gallais, 1984; Riday et al., 2017), and generally reduce disease tolerance (Koffman and Wilsie, 1961). The relative impact of inbreeding depression on the different stages of the alfalfa life cycle has not yet been quantified. Results from previous studies cannot easily be compared among life stages because experiments were done in different environments which can affect inbreeding depression values (Dudash, 1990; Cheptou and Donohue, 2011), distinct varieties or breeding stocks were used, and studies tended to examine a subset of traits and target different portions of the alfalfa life cycle. Determining whether inbreeding depression differentially impacts traits throughout the alfalfa life cycle would inform breeding programs selecting for higher seed or hay yield and could shed further light on the genetic factors underlying inbreeding depression in alfalfa.

We detected inbreeding depression on two seed yield metrics in alfalfa seed production fields, seed set and seeds per pod. When we compared the yield metrics of selfed relative to outcrossed stems, we obtained a 13.4% decrease in the number of seeds per pod and a 15% reduction in seed set per stem corresponding to a value of inbreeding depression of 0.13 for seeds per pod and 0.15 for seed set. We did not detect inbreeding depression on the number of pods set per raceme and average seed weight. A reduction in seeds per pod following selfing has been previously shown to result from pollen tubes reaching and fertilizing fewer ovules, with up to 10 ovules being reached with outcrossing and no more than five ovules with selfing (Viands et al., 1988). While the first four ovules were regularly reached following selfing and outcrossing, only 28% of the first four ovules were fertilized following selfing compared to 80% following outcrossing (Viands et al., 1988). More frequent post-fertilization ovule abortion also plays a role, with 34% of ovules being aborted 48 h after fertilization for selfing relative to 7% with outcrossing (Cooper and Brink, 1940; Viands et al., 1988). Given the inbreeding depression on seeds per pod and seed set, and the increase in the coefficient of inbreeding of the progeny with increased selfing rates, we expected seeds per pod and seed set to decrease as the field selfing rate increased. However, seed set did not change significantly with an increased field selfing rate. Field selfing rate only negatively impacted seed set for outcrossed stems in CEV. Moreover, field selfing rate had little impact on seeds per pod, which was more affected by the number of racemes on a stem. To better understand these patterns, we first examine the modes of selfing occurring in alfalfa seed production fields.

Autogamous, or within-flower selfing, and geitonogamous, or among-flower selfing, represent the two main modes of selfing in plants (Lloyd, 1992). Autonomous autogamy occurs in the absence of pollinators and provides reproductive assurance (Lloyd, 1992; Kalisz et al., 2004). In alfalfa, self-tripping is the mechanism for autonomous autogamy, and the tripping rate can be affected by environmental factors such as temperature (Bohart, 1957; Breazeale et al., 2008; Brunet and Stewart, 2010; Brunet et al., 2019). With autonomous autogamy and self-tripping, we expect only self-fertilized seeds within a pod. However, both self‐ and cross-pollinated seeds typically occur within pods, suggesting that self-tripping plays a minor role in the production of self-pollinated seeds in alfalfa (Bradner and Frakes, 1964; Pankiw and Bolton, 1965; Knapp and Teuber, 1993). Moreover, self-tripping provides low seed set in alfalfa, and good seed production requires tripping of the flowers by bees (Bohart, 1957). The presence of both selfed and outcrossed seeds within pods could be explained by geitonogamous selfing, where pollinators move self-pollen between flowers on a plant, or alternatively by facilitated autogamous selfing, where pollinators move self-pollen to stigmas within flowers (Lloyd, 1992; Knapp and Teuber, 1993). While the level of facilitated autogamous selfing is not expected to change with floral display size, the level of geitonogamous selfing has been shown to increase as pollinators visit more flowers in succession on plants with larger floral displays (Harder and Barrett, 1995; Karron et al., 2004). Both honey bees and leafcutting bees, two major managed pollinators in alfalfa seed production fields, visit many flowers in succession on alfalfa plants (Brunet, unpublished data). In both regions, we observed a statistically significant increase in the number of racemes per stem on selfed stems as field selfing rate increased. Field selfing rate explained a higher proportion of the variance in the number of racemes per stem for selfed relative to outcrossed stems. Selfed stems had more racemes per stem in fields with greater selfing rates, a relationship that strongly supports the presence of geitonogamous selfing in alfalfa seed production fields. Geitonogamous selfing in alfalfa can occur when pollinators move pollen among flowers within or among racemes. Both types of pollen transfer have a similar effect and result in an increase in geitonogamous selfing as pollinators visit more flowers in succession on a plant (Brunet and Sweet, 2006).

Because geitonogamous selfing requires pollinators, it does not provide reproductive assurance. Therefore, in the presence of inbreeding depression, geitonogamous selfing should be selected against a population (Lloyd, 1992; Brunet and Sweet, 2006). Geitonogamy is often considered an unavoidable consequence of cross-pollination in plants with large floral displays (Lloyd, 1992; Jarne and Charlesworth, 1993; Brunet and Sweet, 2006). Facilitated autogamy is also likely to occur in alfalfa seed production and may be a consequence of the tripping mechanism, although this hypothesis requires further investigation. Because both geitonogamy and facilitated autogamy only occur when pollinators are present, the great majority of selfing in alfalfa results from interactions with pollinators and we expect pollinator-mediated selfing to dominate in alfalfa, including in alfalfa seed production fields. A first corollary to pollinator-mediated selfing is that selfing will always occur when pollinators are used to produce seeds, whether in field or in greenhouse settings and irrespective of the field dimensions. In other words, selfed progeny resulting from geitonogamy or from facilitated autogamy cannot be prevented when bees are used for alfalfa pollination. A second corollary to pollinator-mediated selfing is that increasing pollinator abundance will not reduce selfing. Increasing pollinator abundance would only reduce selfing if it were caused by autonomous autogamy, which provides reproductive assurance when pollen is limiting (Kalisz and Vogler, 2003). A third corollary to pollinator-mediated selfing is that to eliminate selfing in alfalfa will require the selection of self-incompatible varieties. During the process of selection, if bees are used for pollination, the presence of bee-mediated selfing must be taken into consideration. Plants with larger floral displays may set more selfed seeds as a result of geitonogamous selfing and appear more self-compatible while they might not be. This pattern will occur in screened polycrosses (Riday et al., 2017) as well as in large multi-acre pollination (this study) as long as bees are used for seed production. Potential metrics to describe floral display size in alfalfa include the number of racemes per flowering stem, or the total number of open flowers per flowering stem (Brunet et al., 2021).

If we examine the relationships between field selfing rate, the number of racemes per stem, and the four seed yield metrics, overall, the number of racemes per stem had a stronger effect on the distinct seed yield metrics relative to the field selfing rate. Contrary to our expectations, based on inbreeding depression on seed set, increasing field selfing rate did not negatively impact seed set on selfed stems. We observed an increase in the number of racemes per stem for selfed stems in fields with higher selfing rates and an increase in seed set for stems with more racemes per stem for both selfed and outcrossed stems. The increase in seed set due to the greater number of racemes per stem for selfed stems in fields with higher selfing exceeds the decrease in seed set resulting from inbreeding depression. Field selfing rate did negatively affect seed set for outcrossed stems at CEV, when raceme number was kept constant. This pattern could be explained by stronger competition for pollinators by outcrossed stems in fields with higher selfing rate. Plants with larger floral displays are more attractive to bees (Mitchell et al., 2004), and this is the case for alfalfa where plants with greater floral displays received more bee visits and set more seeds (Bauer et al., 2017). If the number of racemes per stem is higher for selfed stems in fields with greater selfing rate, outcrossed stems in those fields must compete more for pollinators, which could explain the decrease in seed set on outcrossed stems as the field selfing rate increases. We detected little impact of field selfing rate on seeds per pod or pods per raceme, after keeping the number of racemes constant. Seeds per pod decreased as the number of racemes increased for both selfed and outcrossed stems, while pods per raceme increased for outcrossed stems at CEV. The variation in the number of racemes per stem had more impact on the seed yield metrics than the field selfing rate.

## Conclusion

Selfing was detected in each seed production field and varied among fields. The presence of geitonogamous selfing was supported by the increase in the number of racemes per stem for selfed stems in fields with a greater selfing rate. Fields with higher selfing did not have lower seed set. This seed yield metric was more strongly influenced by the number of racemes per stem than by the field selfing rate. However, relative to outcrossing, selfing did create a 15% reduction in seed set. The presence of a notable coefficient of inbreeding for the progeny (seed) in this study, combined with the detection of inbreeding depression on seed germination and other life history traits in previous studies, suggest the potential reduction in future hay yield. Eliminating selfing in alfalfa seed production fields could lead to a 15% increase in seed yield and an increase in future hay yield. Because selfing in alfalfa is mostly pollinator-mediated, eliminating selfing in alfalfa seed production would require the selection of self-incompatible alfalfa varieties. Identifying the mating system of a crop and its prevailing mode of selfing can guide the development of effective strategies to reduce selfing and increase yield.

## Data Availability Statement

The original contributions presented in the study are included in the article/Supplementary Material, further inquiries can be directed to the corresponding author.

## Author Contributions

JB conceived and acquired funding for the study and supervised the project. MD and JB performed the statistical analyses on the data and wrote and revised the manuscript. LL adjusted some of the backward primers to fit the multiplex PCR. HR supervised the microsatellite work for this project and provided comments on the manuscript. All authors contributed to the article and approved the submitted version.

### Conflict of Interest

The authors declare that the research was conducted in the absence of any commercial or financial relationships that could be construed as a potential conflict of interest.

## Footnotes

^1^https://bioinfo.ut.ee/primer3
