# Supplementary material for: Self-Fertilization, Inbreeding, and Yield in Alfalfa Seed Production
Source: Front Plant Sci. 2021 Jul 6;12:700708. doi: 10.3389/fpls.2021.700708 (PMC8290836; doi:10.3389/fpls.2021.700708)
Supplement: Supplementary file 1 [file Data_Sheet_1.pdf]

Supplementary Material

Supplementary Table 1. The total number of alleles, the average number of alleles per field (± SEM) and the polymorphic information content (PIC) per locus, for parents and progeny.

	Total alleles		Average no alleles per field		PIC	
Locus	Parents N=1274	Progeny N=10,190	Parents N=32 fields	Progeny N=32 fields	Parents N=1274	Progeny N=10,190
BE323955	13	14	9.50 ± 0.22	9.31 ± 0.11	0.82	0.82
AW690665	7	10	4.22 ± 0.07	4.38 ± 0.08	0.76	0.76
BI86	12	15	9.19 ± 0.25	9.03 ± 0.18	0.75	0.75
AW170	9	10	5.03 ± 0.19	5.02 ± 0.15	0.69	0.71
AW658684	7	10	4.34 ± 0.16	4.25 ± 0.13	0.52	0.54
BE119	6	8	4.88 ± 0.07	4.84 ± 0.05	0.77	0.77
BE119-B	10	13	2.88 ± 0.25	3.92 ± 0.21	0.19	0.12
AW235	6	8	4.31 ± 0.08	4.45 ± 0.07	0.68	0.71
BI131	9	10	5.19 ± 0.12	5.36 ± 0.10	0.73	0.75
BI28	16	23	8.78 ± 0.20	8.78 ± 0.16	0.82	0.82
RCS5565	4	4	3.09 ± 0.07	3.07 ± 0.04	0.61	0.61
BG280	9	9	5.22 ± 0.17	5.41 ± 0.11	0.62	0.64
BG222	6	6	3.16 ± 0.09	3.56 ± 0.10	0.64	0.65
BI68	4	3	1.56 ± 0.10	1.59 ± 0.08	0.37	0.39
BG234-left	13	13	7.81 ± 0.26	7.54 ± 0.19	0.63	0.64
BG234-right	7	9	1.69 ± 0.31	2.24 ± 0.26	0.04	0.04
Mean	8.6	10.3	5.05	5.17	0.60	0.61
SEM	0.9	1.2	0.63	0.59	0.06	0.06

SEM is standard error of the mean

Supplementary Table 2. Field selfing rate, field genetic diversity and inbreeding coefficient (Fz) of parents and progeny.

Field		Parents					Progeny				
Name	Selfing Rate	N	Alleles	H_O	H_E	F_Z	N	Alleles*	H_O	H_E	F_Z
CEV_03	9.1	40	5.25 ± 0.79	0.612 ± 0.085	0.593 ± 0.068	0.212	320	5.86 ± 0.73	0.732 ± 0.071	0.639 ± 0.061	0.254
CEV_10	9.1	40	4.75 ± 0.66	0.590 ± 0.084	0.572 ± 0.067	0.175	320	5.08 ± 0.62	0.740 ± 0.072	0.642 ± 0.061	0.187
CEV_04	9.4	40	5.19 ± 0.70	0.595 ± 0.083	0.586 ± 0.067	0.257	320	4.85 ± 0.59	0.725 ± 0.068	0.633 ± 0.058	0.256
CEV_08	10.9	40	5.38 ± 0.81	0.607 ± 0.084	0.591 ± 0.067	0.239	320	5.27 ± 0.69	0.739 ± 0.069	0.646 ± 0.059	0.232
CEV_07	11.6	40	4.94 ± 0.72	0.593 ± 0.089	0.576 ± 0.072	0.209	320	5.39 ± 0.61	0.737 ± 0.070	0.645 ± 0.060	0.234
CEV_02	12.5	40	5.06 ± 0.65	0.603 ± 0.084	0.587 ± 0.069	0.279	320	5.17 ± 0.61	0.735 ± 0.070	0.642 ± 0.060	0.247
CEV_05	14.1	40	4.94 ± 0.69	0.617 ± 0.087	0.588 ± 0.069	0.229	320	4.94 ± 0.64	0.733 ± 0.070	0.640 ± 0.060	0.235
CEV_01	17.2	40	5.19 ± 0.68	0.607 ± 0.089	0.588 ± 0.070	0.194	320	5.21 ± 0.61	0.731 ± 0.068	0.639 ± 0.058	0.250
CEV_09	20.6	40	5.38 ± 0.63	0.608 ± 0.082	0.606 ± 0.062	0.251	320	5.16 ± 0.56	0.730 ± 0.064	0.642 ± 0.055	0.263
CEV average	12.7 ± 1.33	40	5.12 ± 0.07	0.604 ± 0.003	0.587 ± 0.003	0.227 ± 0.011	320	5.21 ± 0.10	0.734 ± 0.002	0.641 ± 0.001	0.240 ± 0.008
IMP_05	8.1	40	4.81 ± 0.61	0.728 ± 0.064	0.634 ± 0.054	0.270	320	4.99 ± 0.58	0.733 ± 0.065	0.640 ± 0.054	0.244
IMP_08	8.1	40	5.00 ± 0.73	0.719 ± 0.070	0.625 ± 0.059	0.263	320	4.98 ± 0.60	0.728 ± 0.069	0.633 ± 0.059	0.241
IMP_07	8.8	40	4.44 ± 0.52	0.716 ± 0.068	0.624 ± 0.057	0.277	320	4.97 ± 0.58	0.739 ± 0.069	0.643 ± 0.058	0.236
IMP_09	10.0	40	5.00 ± 0.66	0.714 ± 0.070	0.621 ± 0.060	0.278	320	4.81 ± 0.56	0.715 ± 0.070	0.624 ± 0.061	0.257
IMP_11	12.2	40	4.38 ± 0.58	0.746 ± 0.070	0.640 ± 0.058	0.147	320	4.75 ± 0.59	0.738 ± 0.068	0.641 ± 0.058	0.177
IMP_12	12.5	40	5.06 ± 0.65	0.735 ± 0.065	0.637 ± 0.055	0.260	319	4.99 ± 0.59	0.744 ± 0.066	0.644 ± 0.055	0.219
IMP_06	12.8	40	5.31 ± 0.69	0.721 ± 0.068	0.630 ± 0.057	0.283	320	5.29 ± 0.62	0.732 ± 0.068	0.642 ± 0.057	0.253
IMP_03	13.1	40	5.00 ± 0.62	0.733 ± 0.065	0.637 ± 0.054	0.265	320	5.17 ± 0.58	0.730 ± 0.063	0.640 ± 0.053	0.262
IMP_02	13.8	40	4.81 ± 0.69	0.725 ± 0.076	0.628 ± 0.065	0.181	320	5.20 ± 0.63	0.737 ± 0.074	0.642 ± 0.064	0.226
IMP_10	14.7	40	5.06 ± 0.76	0.728 ± 0.072	0.632 ± 0.062	0.202	320	5.04 ± 0.70	0.724 ± 0.071	0.634 ± 0.062	0.246
IMP_04	17.2	40	4.56 ± 0.56	0.720 ± 0.061	0.629 ± 0.051	0.271	320	5.03 ± 0.56	0.715 ± 0.061	0.630 ± 0.052	0.292
IMP_01	30.0	40	4.56 ± 0.55	0.743 ± 0.067	0.642 ± 0.056	0.173	320	4.75 ± 0.54	0.753 ± 0.067	0.652 ± 0.056	0.192
IMP average	13.4 ± 1.70	40	4.83 ± 0.08	0.727 ± 0.003	0.632 ± 0.002	0.239 ± 0.014	320	5.00 ± 0.05	0.732 ± 0.003	0.639 ± 0.002	0.237 ± 0.009
PNW_02	5.3	40	5.50 ± 0.77	0.730 ± 0.069	0.639 ± 0.060	0.231	320	5.60 ± 0.72	0.757 ± 0.071	0.658 ± 0.061	0.154
PNW_07	5.8	39	5.63 ± 0.75	0.736 ± 0.068	0.649 ± 0.059	0.247	311	5.50 ± 0.66	0.734 ± 0.071	0.652 ± 0.063	0.198

PNW_10	7.5	40	5.06 ± 0.75	0.749 ± 0.075	0.646 ± 0.064	0.163	320	5.36 ± 0.63	0.743 ± 0.073	0.655 ± 0.064	0.227
PNW_08	8.7	36	5.81 ± 0.71	0.739 ± 0.069	0.652 ± 0.059	0.274	288	5.74 ± 0.62	0.745 ± 0.070	0.659 ± 0.061	0.242
PNW_01	9.4	40	5.31 ± 0.68	0.738 ± 0.069	0.651 ± 0.060	0.222	320	5.58 ± 0.63	0.754 ± 0.068	0.644 ± 0.059	0.211
PNW_11	9.4	40	4.94 ± 0.68	0.741 ± 0.076	0.641 ± 0.065	0.174	320	5.17 ± 0.59	0.738 ± 0.073	0.649 ± 0.064	0.181
PNW_03	11.3	40	4.88 ± 0.66	0.745 ± 0.073	0.651 ± 0.064	0.197	320	5.06 ± 0.62	0.751 ± 0.072	0.659 ± 0.063	0.212
PNW_04	11.5	39	4.56 ± 0.58	0.722 ± 0.073	0.632 ± 0.063	0.201	312	4.64 ± 0.58	0.729 ± 0.073	0.641 ± 0.064	0.181
PNW_09	12.2	40	5.31 ± 0.69	0.738 ± 0.068	0.649 ± 0.058	0.233	320	5.34 ± 0.58	0.735 ± 0.071	0.648 ± 0.062	0.252
PNW_05	15.6	40	5.75 ± 0.69	0.732 ± 0.069	0.639 ± 0.059	0.276	320	5.67 ± 0.63	0.747 ± 0.069	0.652 ± 0.059	0.242
PNW_06	16.9	40	4.88 ± 0.66	0.724 ± 0.072	0.634 ± 0.062	0.243	320	4.92 ± 0.58	0.732 ± 0.072	0.644 ± 0.063	0.228
PNW average	10.3 ± 1.11	39	5.24 ± 0.12	0.736 ± 0.002	0.644 ± 0.002	0.224 ± 0.011	316	5.33 ± 0.10	0.742 ± 0.003	0.653 ± 0.002	0.212 ± 0.009

Genetic diversity is described by the mean number of alleles per locus per field and by the mean observed (Ho) and mean expected (H_E) heterozygosity per locus per field (mean ± SEM). SEM is the standard error of the mean. *The allele number in the progeny is corrected for sample size. The variable N represents the number of maternal plants or the number of progeny (seeds) genotyped per field. Within each region the fields are presented in order of increasing selfing rate. The mean ± SEM is also presented for each variable over each region.

Supplementary Table 3. Analysis of molecular variance (AMOVA) within and among alfalfa seed-production fields and among regions.

Source	df	SS	MS	Variance	% Total Variance
Within fields	81024	948179131.7	11702.4	11702.4	92.30
Among fields	464	5795045.6	12489.3	4.9	0.04
Among regions	32	52857857.4	1651808.0	971.6	7.66

AMOVA was calculated using the genotypes of the 1274 maternal alfalfa plants at 16 SSR loci. The software package 'polygene' was used with the weight genotype method and the stepwise mutation model.

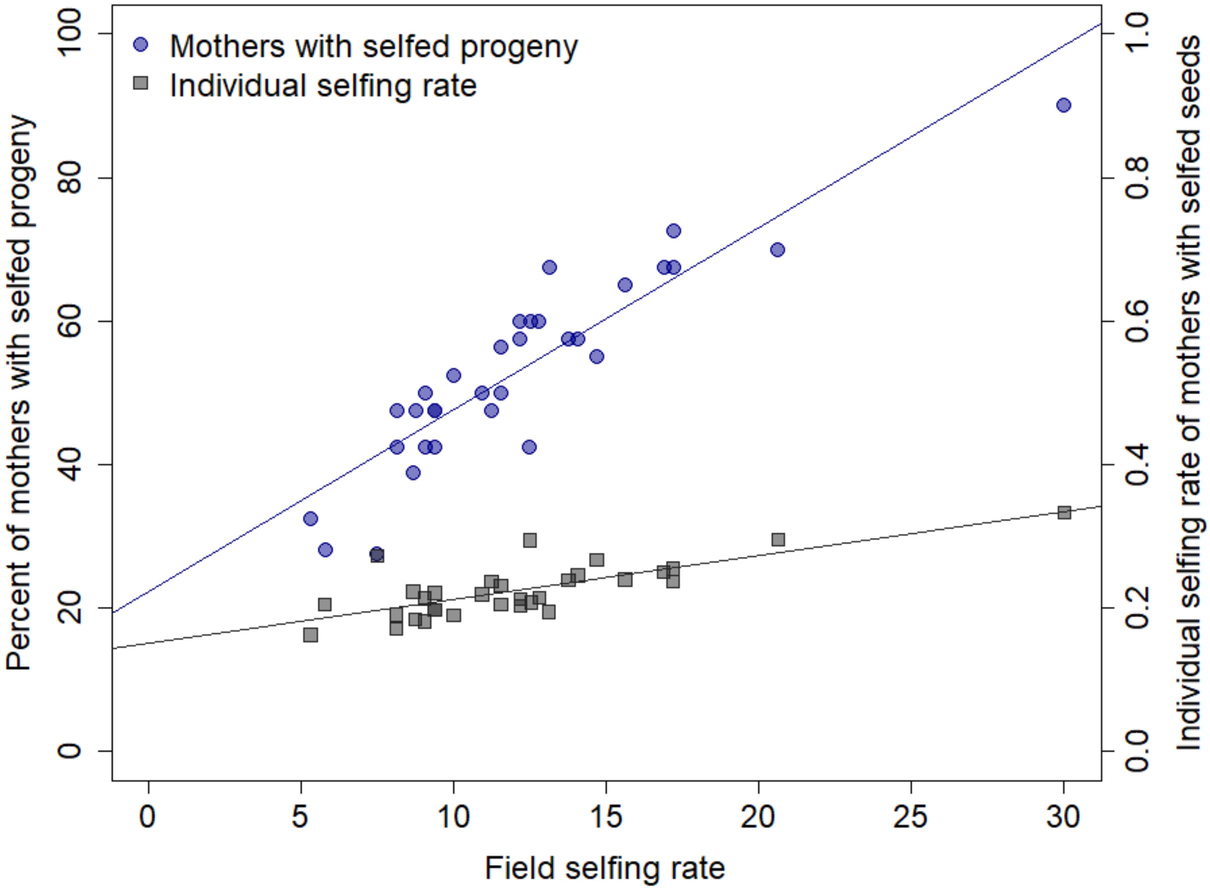

Supplementary Figure 1. Field and individual selfing rates. Single regressions indicated an increase in the percent of mothers with selfed progeny (Y = 2.5x + 22.35, $R^2 = 0.81$, $F_{1,30} = 132.9$, $P < 0.001$) (blue circles, left axis) or the proportion of selfed seeds for individual mothers (individual selfing rate) (Y = 0.006x + 0.15, $R^2 = 0.563$; $F_{1,30} = 40.94$, $P < 0.001$) (gray squares, right axis) with increasing field selfing rate.

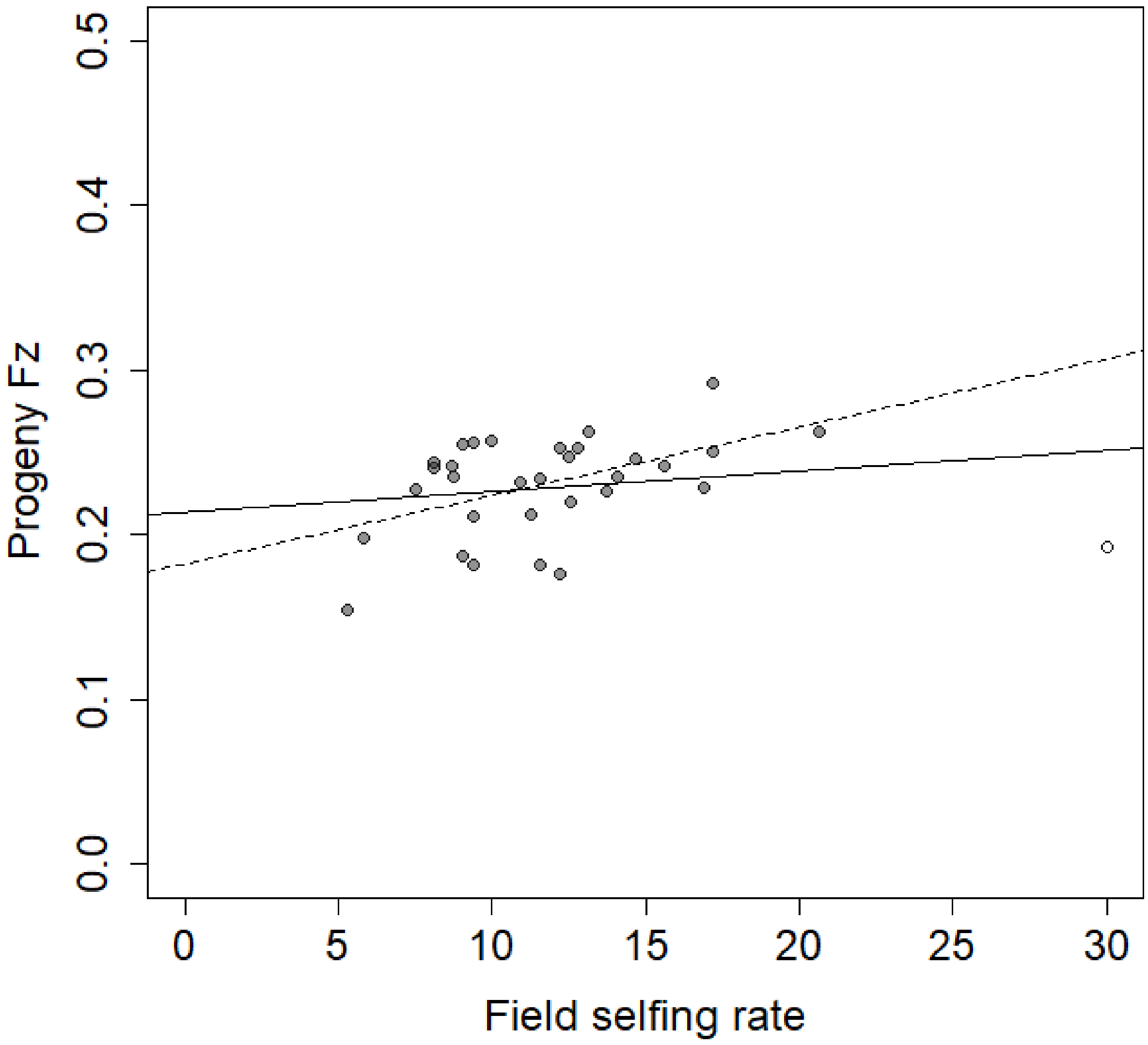

Supplementary Figure 2. Field selfing rate and the progeny inbreeding coefficient (F_Z). Single regressions indicated an increase in progeny F_Z with field selfing rate when the field with the highest selfing rate (open circle, IMP_01) was excluded from the analysis (Y = 0.004x + 0.18, $R^2 = 0.21$, $F_{1,29} = 8.75$, P = 0.006) (dashed line) but not when it was included (Y = 0.001x + 0.21, $R^2 = 0.005$, $F_{1,30} = 1.16$, P = 0.29) (solid line).

Supplementary Data Characteristics of stems

Region	Field	Stem #	Pl Self R	MW 10Seeds	MSW	# Rac	TOT SW	TOTSEED	Pods/Rac	Seeds/Rac	Seeds/Pod	self.out	Field SR
SJV	1	1	0.5	18.5	1.85	24	2197.3	1185.6	6	49.4	8.2	self	17.19
SJV	1	6	0.125	22.3	2.23	30	2428.7	1090.7	5.4	36.4	6.7	self	17.19
SJV	1	7	0	27.1	2.71	9	852.3	314.5	5	34.9	7	out	17.19
SJV	1	8	0.375	20.5	2.05	33	2792.7	1362.3	6.6	41.3	6.3	self	17.19
SJV	1	9	0.125	21.1	2.11	9	879.9	417.7	5.2	46.4	8.9	self	17.19
SJV	1	10	0.5	19.2	1.92	17	2495.5	1299.7	9	76.5	8.5	self	17.19
SJV	1	17	0	21.7	2.17	4	548.8	253.3	11	63.3	5.8	out	17.19
SJV	1	18	0.25	22.3	2.23	22	1150.4	515.9	5.4	23.4	4.3	self	17.19
SJV	1	23	0	27.2	2.72	11	1170	430.7	4.8	39.2	8.2	out	17.19
SJV	1	26	0.25	27.5	2.75	46	2365	859	5.4	18.7	3.5	self	17.19
SJV	1	32	0.375	21.1	2.11	11	1345.6	638.7	10.6	58.1	5.5	self	17.19
SJV	1	33	0.625	23.9	2.39	16	1629.7	680.9	9	42.6	4.7	self	17.19
SJV	1	35	0	19.7	1.97	26	2490.6	1262.1	6	48.5	8.1	out	17.19
SJV	1	36	0.25	26.6	2.66	10	1503.4	564.5	10	56.4	5.6	self	17.19
SJV	1	37	0	24.8	2.48	29	1812.2	731.7	3.4	25.2	7.4	out	17.19
SJV	1	39	0.125	22.9	2.29	16	1297.5	565.8	3.8	35.4	9.3	self	17.19
SJV	1	42	0	29.9	2.99	49	2705.8	904.9	7	18.5	2.6	out	17.19
SJV	1	45	0.375	25.2	2.52	7	517.7	205.4	5.8	29.3	5.1	self	17.19
SJV	1	46	0.25	23.9	2.39	17	966.7	404.5	5.4	23.8	4.4	self	17.19
SJV	1	50	0	19.9	1.99	8	1082.6	544	6	68	11.3	out	17.19
SJV	2	2	0.25	21.4	2.14	14	804.8	375.5	7.8	26.8	3.4	self	12.5
SJV	2	3	0.125	29.6	2.96	12	696.6	235.1	4.6	19.6	4.3	self	12.5
SJV	2	5	0	21.2	2.12	7	256.8	120.9	4.8	17.3	3.6	out	12.5
SJV	2	6	0	19.9	1.99	11	345.1	173.4	4	15.8	3.9	out	12.5
SJV	2	7	0.75	20.9	2.09	16	802.2	384.4	6	24	4	self	12.5
SJV	2	8	0.125	23.7	2.37	16	641.8	270.4	5.4	16.9	3.1	self	12.5
SJV	2	11	0.375	30.9	3.09	14	1527.6	494.9	5.6	35.4	6.3	self	12.5
SJV	2	12	0	21.5	2.15	24	1281.5	595.1	7.2	24.8	3.4	out	12.5
SJV	2	13	0	24	2.4	18	1439.1	598.8	8.2	33.3	4.1	out	12.5
SJV	2	16	0.125	20.1	2.01	27	1365.5	680.5	8.4	25.2	3	self	12.5
SJV	2	17	0	25.2	2.52	18	690.6	274.4	6.6	15.2	2.3	out	12.5

SJV	2	19	0.5	26.4	2.64	5	482.7	182.6	4	36.5	9.1	self	12.5
SJV	2	20	0	29	2.9	15	737.7	254.7	3.4	17	5	out	12.5
SJV	2	24	0	27.3	2.73	25	2563.3	938.9	9.2	37.6	4.1	out	12.5
SJV	2	44	0.25	28	2.8	66	4150.2	1480.5	7	22.4	3.2	self	12.5
SJV	2	45	0	27.2	2.72	26	1772.2	650.7	6.8	25	3.7	out	12.5
SJV	2	48	0	31.9	3.19	14	1756.5	550.6	7	39.3	5.6	out	12.5
SJV	2	50	0.25	20.3	2.03	8	563.3	277	9.4	34.6	3.7	self	12.5
SJV	3	1	0	21.3	2.13	65	6754.4	3171.1	8.6	48.8	5.7	out	9.06
SJV	3	2	0	25.2	2.52	25	2461.8	976.9	7.6	39.1	5.1	out	9.06
SJV	3	4	0	23.7	2.37	13	914.4	385.3	5.4	29.6	5.5	out	9.06
SJV	3	5	0	26.2	2.62	17	3193.6	1220.5	11.6	71.8	6.2	out	9.06
SJV	3	12	0.125	26.9	2.69	18	1229.5	457.1	6.4	25.4	4	self	9.06
SJV	3	13	0.125	21.5	2.15	16	1439.3	669.4	7	41.8	6	self	9.06
SJV	3	14	0	31.1	3.11	20	595.1	191.6	3.4	9.6	2.8	out	9.06
SJV	3	15	0	30.4	3.04	15	1009	331.5	4.8	22.1	4.6	out	9.06
SJV	3	19	0.125	21.5	2.15	16	1350.2	629	8.4	39.3	4.7	self	9.06
SJV	3	20	0.125	25	2.5	11	1063.4	424.8	5	38.6	7.7	self	9.06
SJV	3	21	0	25	2.5	10	1132	452.2	5.8	45.2	7.8	out	9.06
SJV	3	28	0	27.5	2.75	7	841.4	305.6	7.4	43.7	5.9	out	9.06
SJV	3	29	0.125	23.9	2.39	18	1018.3	425.5	5.2	23.6	4.5	self	9.06
SJV	3	31	0.125	20.9	2.09	25	2102.1	1004.2	6.8	40.2	5.9	self	9.06
SJV	3	32	0.375	29.6	2.96	11	794.6	268.1	5.6	24.4	4.4	self	9.06
SJV	3	46	0	25	2.5	12	1440.6	575.5	9	48	5.3	out	9.06
SJV	3	47	0.125	23	2.3	15	1949	846.2	10.4	56.4	5.4	self	9.06
SJV	3	48	0	23.2	2.32	26	2589.4	1114.5	7.4	42.9	5.8	out	9.06
SJV	3	49	0	22.1	2.21	17	2006.4	906.5	8.4	53.3	6.3	out	9.06
SJV	3	50	0	24	2.4	9	816.8	339.9	4.6	37.8	8.2	out	9.06
SJV	4	1	0.25	28.1	2.81	8	940.6	334.7	11.8	41.8	3.5	self	9.38
SJV	4	2	0.125	26.6	2.66	15	111.7	42	8.4	2.8	0.3	self	9.38
SJV	4	3	0	25.6	2.56	21	2055.5	801.9	6.8	38.2	5.6	out	9.38
SJV	4	4	0.125	30.9	3.09	12	1745.1	565.4	7.6	47.1	6.2	self	9.38
SJV	4	7	0	31.2	3.12	15	1172.1	375.7	9.4	25	2.7	out	9.38
SJV	4	8	0	24.3	2.43	19	1450	595.9	8.6	31.4	3.6	out	9.38

SJV	4	9	0	24.3	2.43	28	3744.7	1543.1	9.6	55.1	5.7	out	9.38
SJV	4	10	0	23.9	2.39	17	1820.2	762.7	5.4	44.9	8.3	out	9.38
SJV	4	13	0	26.2	2.62	50	4849.5	1851	12.6	37	2.9	out	9.38
SJV	4	16	0	29.4	2.94	8	1369.1	465.7	8.8	58.2	6.6	out	9.38
SJV	4	19	0.5	27.2	2.72	15	1192.6	438.5	9	29.2	3.2	self	9.38
SJV	4	20	0.125	27.4	2.74	16	1733.5	633.4	6	39.6	6.6	self	9.38
SJV	4	21	0	25.8	2.58	20	2228.4	862.6	6	43.1	7.2	out	9.38
SJV	4	28	0.125	29.9	2.99	13	828	276.9	5.4	21.3	3.9	self	9.38
SJV	4	29	0.125	22.4	2.24	15	1788.2	798.3	10	53.2	5.3	self	9.38
SJV	4	31	0	26.4	2.64	12	1753.9	665.2	6.8	55.4	8.2	out	9.38
SJV	4	38	0	24.8	2.48	24	1776.6	715.4	5.8	29.8	5.1	out	9.38
SJV	4	41	0	25.6	2.56	7	767.2	299.7	6.2	42.8	6.9	out	9.38
SJV	4	42	0.125	24.3	2.43	17	2181.7	899.1	11.4	52.9	4.6	self	9.38
SJV	5	1	0	23.6	2.36	17	1452.4	615.4	10.8	36.2	3.4	out	14.06
SJV	5	2	0.375	19.8	1.98	17	157.6	79.7	9	4.7	0.5	self	14.06
SJV	5	5	0	26.6	2.66	35	2384.8	897.7	8	25.6	3.2	out	14.06
SJV	5	6	0	23.5	2.35	30	1717.1	731.7	6.8	24.4	3.6	out	14.06
SJV	5	7	0.75	21.8	2.18	24	2002.3	917.1	10.2	38.2	3.7	self	14.06
SJV	5	8	0	21.3	2.13	27	2270.5	1067.6	5.6	39.5	7.1	out	14.06
SJV	5	9	0	26.1	2.61	25	1957.6	750	8.6	30	3.5	out	14.06
SJV	5	28	0.25	28.6	2.86	10	1445.1	505.3	11.4	50.5	4.4	self	14.06
SJV	5	29	0	19.8	1.98	18	1172.9	593.4	7.8	33	4.2	out	14.06
SJV	5	30	0.25	23.8	2.38	32	2950.2	1241.3	11.8	38.8	3.3	self	14.06
SJV	5	31	0.25	22.6	2.26	12	1558.8	688.7	10.8	57.4	5.3	self	14.06
SJV	5	32	0	22.3	2.23	19	1040.5	466.6	8.6	24.6	2.9	out	14.06
SJV	5	33	0	20	2	21	2129.6	1064.8	9.2	50.7	5.5	out	14.06
SJV	5	34	0.125	24.5	2.45	21	2022.7	826.7	8	39.4	4.9	self	14.06
SJV	5	35	0.25	26.3	2.63	16	1046.2	398.3	5.6	24.9	4.4	self	14.06
SJV	5	40	0	20.7	2.07	26	1985	957.4	9.4	36.8	3.9	out	14.06
SJV	5	44	0	22.8	2.28	14	1610.2	706.2	5.2	50.4	9.7	out	14.06
SJV	5	47	0	23.2	2.32	38	3729.8	1607.7	9.2	42.3	4.6	out	14.06
SJV	7	1	0.125	22.6	2.26	20	2160.1	955.8	6.4	47.8	7.5	self	11.56
SJV	7	3	0	23.6	2.36	9	631.6	267.2	9.6	29.7	3.1	out	11.56

SJV	7	5	0	22.7	2.27	17	2294.9	1012.5	12.8	59.6	4.7	out	11.56
SJV	7	6	0.25	25.5	2.55	10	666.4	261	5.4	26.1	4.8	self	11.56
SJV	7	10	0.125	26	2.6	7	612.5	235.3	5.4	33.6	6.2	self	11.56
SJV	7	17	0	23.4	2.34	7	901.6	384.8	6.2	55	8.9	out	11.56
SJV	7	18	0	23	2.3	9	1196.1	520.8	9	57.9	6.4	out	11.56
SJV	7	19	0	22.9	2.29	22	2371.9	1034.3	9	47	5.2	out	11.56
SJV	7	21	0.125	27.5	2.75	9	812.4	295.1	7	32.8	4.7	self	11.56
SJV	7	24	0	23.9	2.39	23	2749.5	1152	9.2	50.1	5.4	out	11.56
SJV	7	25	0.125	18.3	1.83	5	567.2	309.9	11	62	5.6	self	11.56
SJV	7	28	0.125	19.9	1.99	12	1360.5	682.5	10	56.9	5.7	self	11.56
SJV	7	30	0.125	31	3.1	19	1162.6	374.6	7.6	19.7	2.6	self	11.56
SJV	7	31	0	23.7	2.37	44	2696	1137.6	9	25.9	2.9	out	11.56
SJV	7	34	0	22.3	2.23	9	591.8	265.4	3.6	29.5	8.2	out	11.56
SJV	7	40	0	27.8	2.78	21	1545	555.1	6.2	26.4	4.3	out	11.56
SJV	7	41	0	25.3	2.53	9	701.1	277.5	5.2	30.8	5.9	out	11.56
SJV	7	42	0.375	23.5	2.35	4	621.8	264.6	13.8	66.1	4.8	self	11.56
SJV	7	44	0	27.2	2.72	10	1231.3	452.7	7.4	45.3	6.1	out	11.56
SJV	7	50	0	23	2.3	6	734.5	318.9	7	53.1	7.6	out	11.56
SJV	8	1	0	24	2.4	30	2724.3	1135.1	12.4	37.8	3.1	out	10.94
SJV	8	2	0.25	20.9	2.09	22	1729.1	826	9.4	37.5	4	self	10.94
SJV	8	4	0	22.5	2.25	25	790	351.6	7.4	14.1	1.9	out	10.94
SJV	8	5	0	25.5	2.55	11	1582.9	619.9	4.4	56.4	12.8	out	10.94
SJV	8	6	0	20.6	2.06	19	2114.6	1028.2	8	54.1	6.8	out	10.94
SJV	8	8	0	25.3	2.53	7	282.6	111.7	5.4	16	3	out	10.94
SJV	8	9	0.25	25.7	2.57	8	706.6	275.3	6.6	34.4	5.2	self	10.94
SJV	8	12	0.125	26.2	2.62	12	660	251.9	6	21	3.5	self	10.94
SJV	8	14	0	23.2	2.32	15	1010.3	436.1	8.6	29.1	3.4	out	10.94
SJV	8	16	0.375	25.5	2.55	27	3142.9	1230.9	11.6	45.6	3.9	self	10.94
SJV	8	18	0	23.2	2.32	18	1637.6	706.9	4.8	39.3	8.2	out	10.94
SJV	8	24	0	21.7	2.17	22	2262.6	1041.1	7	47.3	6.8	out	10.94
SJV	8	28	0.125	21.1	2.11	41	2460.5	1164.3	6.4	28.4	4.4	self	10.94
SJV	8	31	0	25.2	2.52	19	1805.3	715.4	8.6	37.7	4.4	out	10.94
SJV	8	33	0	21.1	2.11	14	1381.8	654.9	8	46.8	5.8	out	10.94

SJV	8	36	0.125	25	2.5	11	838.1	335.7	4.2	30.5	7.3	self	10.94
SJV	8	37	0.375	22.6	2.26	14	2296.1	1014.5	16	72.5	4.5	self	10.94
SJV	8	38	0.25	28.9	2.89	21	1565.1	542.2	7.8	25.8	3.3	self	10.94
SJV	8	39	0.375	25.1	2.51	22	941.6	375.6	4.6	17.1	3.7	self	10.94
SJV	8	45	0.25	21.1	2.11	26	2709.7	1282.2	15.2	49.3	3.2	self	10.94
SJV	9	2	0	24.7	2.47	40	1738	703.6	5.4	17.6	3.3	out	20.63
SJV	9	3	0.25	28.7	2.87	28	2284.7	796.1	8.2	28.4	3.5	self	20.63
SJV	9	4	0.25	24.9	2.49	44	2388.8	958.1	11	21.8	2	self	20.63
SJV	9	6	0.125	25.1	2.51	30	1864.4	741.8	8.6	24.7	2.9	self	20.63
SJV	9	7	0.25	28	2.8	28	1448	517.1	6.8	18.5	2.7	self	20.63
SJV	9	13	0	27.5	2.75	18	1426.3	518	10	28.8	2.9	out	20.63
SJV	9	14	0	26.1	2.61	8	829.3	317.3	7.8	39.7	5.1	out	20.63
SJV	9	16	0	24.3	2.43	24	1749.5	720	9	30	3.3	out	20.63
SJV	9	18	0.25	23.3	2.33	11	924.7	397.4	5.6	36.1	6.5	self	20.63
SJV	9	20	0	26.8	2.68	10	1219	454.9	10.6	45.5	4.3	out	20.63
SJV	9	25	0	26.1	2.61	19	1253.9	480.4	5.6	25.3	4.5	out	20.63
SJV	9	28	0.375	22.8	2.28	14	1036.6	454	9.2	32.4	3.5	self	20.63
SJV	9	29	0.125	22.6	2.26	20	1429.6	631.6	8.8	31.6	3.6	self	20.63
SJV	9	30	0.25	19.4	1.94	16	791.7	408.8	6.8	25.5	3.8	self	20.63
SJV	9	35	0.25	32.6	3.26	22	999.5	306.6	8.8	13.9	1.6	self	20.63
SJV	9	36	0.125	23	2.3	21	2305	1000.7	8	47.7	6	self	20.63
SJV	9	38	0	21.6	2.16	27	2348.9	1087.5	9.6	40.3	4.2	out	20.63
SJV	9	41	0.5	24.5	2.45	14	1077	439	9.6	31.4	3.3	self	20.63
SJV	9	46	0.125	22.9	2.29	14	894	389.8	5.6	27.8	5	self	20.63
SJV	10	6	0	19.8	1.98	7	665.9	335.7	7.4	48	6.5	out	9.06
SJV	10	11	0.125	26.9	2.69	10	829.4	308.3	8.8	30.8	3.5	self	9.06
SJV	10	12	0	17.1	1.71	15	853.3	500	5.2	33.3	6.4	out	9.06
SJV	10	18	0	18.1	1.81	12	850.5	469.9	5.6	39.2	7	out	9.06
SJV	10	20	0	18.5	1.85	18	1025.7	554.4	6.2	30.8	5	out	9.06
SJV	10	21	0	19.4	1.94	7	442.3	227.6	6.6	32.5	4.9	out	9.06
SJV	10	22	0	13.3	1.33	18	1048.6	786.5	6.2	43.7	7	out	9.06
SJV	10	23	0.25	18.4	1.84	5	427.1	232.1	10.2	46.4	4.6	self	9.06
SJV	10	27	0.25	14.6	1.46	9	597.4	410.1	6.8	45.6	6.7	self	9.06

SJV	10	28	0.25	15.6	1.56	9	1031.2	659.6	8.2	73.3	8.9	self	9.06
SJV	10	29	0.125	15.2	1.52	5	452.1	298.1	8.4	59.6	7.1	self	9.06
SJV	10	30	0	28.4	2.84	7	714.1	251.7	4.8	36	7.5	out	9.06
SJV	10	38	0	18	1.8	8	891.4	494.3	5.2	61.8	11.9	out	9.06
SJV	10	44	0	27.8	2.78	9	643.5	231.5	8.4	25.7	3.1	out	9.06
SJV	10	45	0	19.5	1.95	17	984	503.8	5	29.6	5.9	out	9.06
SJV	10	46	0.25	17.5	1.75	16	943.6	538.2	2.8	33.6	12	self	9.06
SJV	10	47	0	19.4	1.94	11	592.7	305.5	7.2	27.8	3.9	out	9.06
SJV	10	48	0.125	15.9	1.59	6	601.1	378.1	12.2	63	5.2	self	9.06
SJV	10	49	0.125	18	1.8	12	767.1	426.2	9	35.5	3.9	self	9.06
SJV	10	50	0	18.6	1.86	20	1196.9	644.6	7.4	32.2	4.4	out	9.06
IMP	1	1	0.125	23.2	2.32	38	2135.4	921.8	5.4	24.3	4.5	self	30
IMP	1	5	0.25	23.2	2.32	12	401.6	173.1	7.8	14.4	1.8	self	30
IMP	1	6	0.125	24.7	2.47	23	416.6	168.9	4.2	7.3	1.7	self	30
IMP	1	8	0.375	27	2.7	32	1963.9	727.4	10.2	22.7	2.2	self	30
IMP	1	11	0	24.9	2.49	9	830.3	333.5	8.2	37.1	4.5	out	30
IMP	1	13	1	25.4	2.54	20	1444.8	568.8	9.2	28.4	3.1	self	30
IMP	1	16	0.375	24.2	2.42	28	587.8	243.2	5.4	8.7	1.6	self	30
IMP	1	20	0.25	21.5	2.15	18	519.8	241.4	8.4	13.4	1.6	self	30
IMP	1	21	0.125	25.1	2.51	14	641.4	255.5	7	18.3	2.6	self	30
IMP	1	22	0.25	18.7	1.87	34	668.6	358.2	5.6	10.5	1.9	self	30
IMP	1	27	0.375	23.4	2.34	15	1301	555.2	9.4	37	3.9	self	30
IMP	1	31	0.5	25.3	2.53	17	852.4	337.4	7.2	19.8	2.8	self	30
IMP	1	32	0.125	24.7	2.47	15	682.8	276.4	8	18.4	2.3	self	30
IMP	1	33	0.625	16.2	1.62	11	420.9	260.4	10.6	23.7	2.2	self	30
IMP	1	43	0.25	22.4	2.24	12	317.5	141.5	8.2	11.8	1.4	self	30
IMP	1	45	0.25	19.1	1.91	10	308.7	161.3	8.8	16.1	1.8	self	30
IMP	1	48	0.125	18.3	1.83	18	180.7	98.9	5.6	5.5	1	self	30
IMP	1	49	0.5	25.2	2.52	13	673	267.1	5.4	20.5	3.8	self	30
IMP	1	50	0.5	22.2	2.22	21	1940.8	875.5	11.2	41.7	3.7	self	30
IMP	2	1	0	23.9	2.39	31	1068.4	447	9	14.4	1.6	out	13.75
IMP	2	2	0	20.3	2.03	11	340	167.8	7	15.3	2.2	out	13.75
IMP	2	4	0.375	28.7	2.87	46	2033.9	707.9	9	15.4	1.7	self	13.75

IMP	2	5	0.375	27.2	2.72	15	435.3	159.8	4.4	10.7	2.4	self	13.75
IMP	2	8	0.375	26.4	2.64	18	921	349.3	7.4	19.4	2.6	self	13.75
IMP	2	11	0	26.4	2.64	15	842.6	318.8	7	21.3	3	out	13.75
IMP	2	12	0	21.7	2.17	7	173.4	79.8	3.6	11.4	3.2	out	13.75
IMP	2	16	0.25	25.3	2.53	16	358.8	141.6	6.4	8.9	1.4	self	13.75
IMP	2	29	0.5	20.8	2.08	16	1082.7	520.5	7.2	32.5	4.5	self	13.75
IMP	2	30	0.375	24.6	2.46	8	436.3	177.4	8.4	22.2	2.6	self	13.75
IMP	2	36	0	27.3	2.73	6	298.2	109.2	6	18.2	3	out	13.75
IMP	2	37	0	21.9	2.19	21	1341.9	611.8	8.4	29.1	3.5	out	13.75
IMP	2	42	0	25.3	2.53	32	1658.7	656.5	8.2	20.5	2.5	out	13.75
IMP	2	43	0	19.7	1.97	23	1173.5	596.7	8.2	25.9	3.2	out	13.75
IMP	2	47	0	25.1	2.51	14	300.2	119.6	6.8	8.5	1.3	out	13.75
IMP	2	48	0.25	23.6	2.36	26	816.8	345.6	6.2	13.3	2.1	self	13.75
IMP	2	50	0.125	25	2.5	8	222.3	89	4	11.1	2.8	self	13.75
IMP	3	1	0.125	21.6	2.16	9	459	212.2	6.6	23.6	3.6	self	13.13
IMP	3	2	0	25.9	2.59	16	863.8	333.5	6.8	20.8	3.1	out	13.13
IMP	3	9	0	20	2	16	699.4	350.3	5	21.9	4.4	out	13.13
IMP	3	12	0.125	27.7	2.77	13	241.5	87.3	6.6	6.7	1	self	13.13
IMP	3	13	0.125	15.6	1.56	12	539.1	346.3	6.8	28.9	4.2	self	13.13
IMP	3	17	0.375	26	2.6	10	481.3	185.1	4.8	18.5	3.9	self	13.13
IMP	3	18	0.375	28	2.8	8	346.2	123.6	6.4	15.5	2.4	self	13.13
IMP	3	21	0	14.5	1.45	10	576	397.2	7	39.7	5.7	out	13.13
IMP	3	29	0	16.8	1.68	19	701.6	416.8	6.8	21.9	3.2	out	13.13
IMP	3	30	0	22.1	2.21	18	250.6	113.4	5	6.3	1.3	out	13.13
IMP	3	31	0.25	22.6	2.26	9	545.7	241.8	4.2	26.9	6.4	self	13.13
IMP	3	32	0	25.3	2.53	8	323.7	127.8	5	16	3.2	out	13.13
IMP	3	33	0.25	20.3	2.03	7	539.1	265.6	3.8	37.9	10	self	13.13
IMP	3	34	0	30.3	3.03	5	172.8	57	2.8	11.4	4.1	out	13.13
IMP	3	43	0.125	18.9	1.89	15	657.9	348.7	6.8	23.2	3.4	self	13.13
IMP	3	44	0.125	32.1	3.21	10	517.6	161.1	6.2	16.1	2.6	self	13.13
IMP	3	49	0	15.6	1.56	10	271	173.7	5.2	17.4	3.3	out	13.13
IMP	3	50	0.25	24.7	2.47	12	985.2	399.4	7.8	33.3	4.3	self	13.13
IMP	4	2	0.375	21.8	2.18	14	679.9	312.4	5.6	22.3	4	self	17.19

IMP	4	6	0.25	21.8	2.18	11	779.6	358.2	7	32.6	4.7	self	17.19
IMP	4	8	0.25	29.5	2.95	20	1636.8	555.5	9.2	27.8	3	self	17.19
IMP	4	10	0.25	24.8	2.48	12	880.7	354.6	7.8	29.6	3.8	self	17.19
IMP	4	11	0	28.5	2.85	6	655.7	230.3	14	38.4	2.7	out	17.19
IMP	4	13	0.625	23	2.3	12	952.9	414.3	13.4	34.5	2.6	self	17.19
IMP	4	18	0.25	22.5	2.25	7	370.2	164.8	7.2	23.5	3.3	self	17.19
IMP	4	19	0.375	25.5	2.55	7	297.8	116.9	10.6	16.7	1.6	self	17.19
IMP	4	20	0	23.8	2.38	35	1148.6	483.3	10.4	13.8	1.3	out	17.19
IMP	4	21	0.125	27	2.7	11	1072.9	397.9	9.2	36.2	3.9	self	17.19
IMP	4	25	0.25	27.1	2.71	12	602	221.9	7	18.5	2.6	self	17.19
IMP	4	26	0	27.7	2.77	12	700.3	252.8	5.8	21.1	3.6	out	17.19
IMP	4	30	0.125	29.2	2.92	15	1138.8	389.6	7	26	3.7	self	17.19
IMP	4	31	0	20.4	2.04	10	1044.9	511.4	9.2	51.1	5.6	out	17.19
IMP	4	32	0.125	22.3	2.23	11	216.7	97.2	5	8.8	1.8	self	17.19
IMP	4	36	0	25.1	2.51	11	656.8	261.3	6.8	23.8	3.5	out	17.19
IMP	4	38	0	28.7	2.87	15	1387.9	483	8.8	32.2	3.7	out	17.19
IMP	4	40	0	22.2	2.22	4	515.8	232.7	12	58.2	4.8	out	17.19
IMP	4	45	0	19.1	1.91	11	1482.9	775	11.4	70.5	6.2	out	17.19
IMP	4	46	0.375	22.4	2.24	8	686.9	307.1	6.8	38.4	5.6	self	17.19
IMP	5	2	0.125	27.2	2.72	16	520.1	191	5.6	11.9	2.1	self	8.13
IMP	5	4	0.375	20.8	2.08	10	634.6	305.1	7.6	30.5	4	self	8.13
IMP	5	7	0	14.6	1.46	10	244.6	167.9	3.4	16.8	4.9	out	8.13
IMP	5	8	0.125	20.6	2.06	15	566.8	275.1	5	18.3	3.7	self	8.13
IMP	5	11	0.25	22.5	2.25	4	162.3	72	5.5	18	3.3	self	8.13
IMP	5	17	0	28.7	2.87	12	1132.8	394.7	10.4	32.9	3.2	out	8.13
IMP	5	26	0.125	28.2	2.82	12	298	105.8	5.8	8.8	1.5	self	8.13
IMP	5	27	0	23.6	2.36	18	982.6	415.8	9.2	23.1	2.5	out	8.13
IMP	5	34	0	27.2	2.72	12	254	93.5	3	7.8	2.6	out	8.13
IMP	5	40	0.125	18	1.8	11	1285.9	713.1	10.2	64.8	6.4	self	8.13
IMP	5	41	0.125	30.4	3.04	10	579.7	190.9	5.2	19.1	3.7	self	8.13
IMP	5	42	0	29.6	2.96	11	747.5	252.2	6.6	22.9	3.5	out	8.13
IMP	5	43	0	18.1	1.81	5	664.5	367.8	8.6	73.6	8.6	out	8.13
IMP	5	44	0.25	28	2.8	7	301.5	107.8	5	15.4	3.1	self	8.13

IMP	5	45	0.25	24.8	2.48	10	504	203	6	20.3	3.4	self	8.13
IMP	5	46	0.125	28.9	2.89	7	357.4	123.5	4.6	17.6	3.8	self	8.13
IMP	5	47	0.25	17.3	1.73	13	1207.8	699.5	12	53.8	4.5	self	8.13
IMP	5	48	0	18.3	1.83	18	1798.4	984.5	10.8	54.7	5.1	out	8.13
IMP	5	49	0	32.2	3.22	8	962.7	299.3	8.4	37.4	4.5	out	8.13
IMP	5	50	0	23.3	2.33	12	660.9	283.2	4.8	23.6	4.9	out	8.13
IMP	6	1	0.375	28	2.8	10	371.6	132.6	4.8	13.3	2.8	self	12.81
IMP	6	2	0.125	18.4	1.84	8	206.5	112	9	14	1.6	self	12.81
IMP	6	3	0.375	26.5	2.65	20	1555.3	586.2	6.2	29.3	4.7	self	12.81
IMP	6	17	0.125	17.1	1.71	14	123.4	72.2	6.6	5.2	0.8	self	12.81
IMP	6	20	0	25.8	2.58	10	392.3	152.3	6.6	15.2	2.3	out	12.81
IMP	6	29	0.125	24.9	2.49	10	767.2	308.5	10	30.9	3.1	self	12.81
IMP	6	30	0	23.8	2.38	9	222.8	93.5	4.6	10.4	2.3	out	12.81
IMP	6	31	0.125	26.3	2.63	4	112	42.6	5.3	10.7	2	self	12.81
IMP	6	32	0.125	24.6	2.46	20	1091.1	444.1	8.6	22.2	2.6	self	12.81
IMP	6	33	0	26.2	2.62	9	281.8	107.6	4.6	12	2.6	out	12.81
IMP	6	34	0	26.3	2.63	3	112.9	42.9	5	14.3	2.9	out	12.81
IMP	6	35	0.25	28	2.8	4	516.1	184.1	8.8	46	5.2	self	12.81
IMP	6	36	0	23.7	2.37	5	340.5	143.5	8.6	28.7	3.3	out	12.81
IMP	6	37	0.5	27.8	2.78	15	267.6	96.1	3.4	6.4	1.9	self	12.81
IMP	6	38	0.125	26.4	2.64	7	190	72	5	10.3	2.1	self	12.81
IMP	6	39	0	25.6	2.56	9	331.9	129.5	6	14.4	2.4	out	12.81
IMP	6	42	0.25	22.6	2.26	4	169.8	75	5.5	18.8	3.4	self	12.81
IMP	6	43	0.25	23.9	2.39	11	288.1	120.4	4.8	10.9	2.3	self	12.81
IMP	6	44	0.125	25.7	2.57	3	235.7	91.7	8	30.6	3.8	self	12.81
IMP	6	45	0	33	3.3	6	400.7	121.4	8	20.2	2.5	out	12.81
IMP	6	46	0	32	3.2	9	461.5	144.1	7	16	2.3	out	12.81
IMP	6	47	0	20.6	2.06	13	443.3	214.8	4.8	16.5	3.4	out	12.81
IMP	6	48	0	24.7	2.47	7	254.8	103.2	4.6	14.7	3.2	out	12.81
IMP	6	49	0.25	25.8	2.58	10	507	196.3	8	19.6	2.5	self	12.81
IMP	6	50	0	23.4	2.34	21	906.6	387.4	5.6	18.4	3.3	out	12.81
IMP	7	1	0	23.9	2.39	13	806.7	338	6.8	26	3.8	out	8.75
IMP	7	2	0	31.2	3.12	10	233.7	74.9	4.8	7.5	1.6	out	8.75

IMP	7	7	0.125	26.4	2.64	11	620.3	235	5.6	21.4	3.8	self	8.75
IMP	7	8	0.25	25.5	2.55	10	411.3	161.5	5.6	16.2	2.9	self	8.75
IMP	7	16	0	23.4	2.34	12	781.8	333.6	9	27.8	3.1	out	8.75
IMP	7	17	0	24.5	2.45	9	468.5	191.2	5.6	21.2	3.8	out	8.75
IMP	7	18	0	24.4	2.44	15	323.4	132.4	4.4	8.8	2	out	8.75
IMP	7	21	0	21.3	2.13	9	712.1	333.8	9.8	37.1	3.8	out	8.75
IMP	7	22	0.375	13.8	1.38	14	93.4	67.7	4.8	4.8	1	self	8.75
IMP	7	28	0.125	23.3	2.33	4	163.9	70.4	3.75	17.6	4.7	self	8.75
IMP	7	29	0	24.9	2.49	12	571.2	229.7	5.6	19.1	3.4	out	8.75
IMP	7	30	0	25.2	2.52	16	1025.4	406.9	8.4	25.4	3	out	8.75
IMP	7	31	0.125	25.3	2.53	9	533.6	210.9	5.4	23.4	4.3	self	8.75
IMP	7	34	0	26.5	2.65	14	557.4	210.3	6.6	15	2.3	out	8.75
IMP	7	35	0	25	2.5	20	985.7	393.8	5.4	19.7	3.6	out	8.75
IMP	7	40	0	25.6	2.56	23	1329	518.5	9	22.5	2.5	out	8.75
IMP	7	41	0	22	2.2	16	1284.5	584.7	6.4	36.5	5.7	out	8.75
IMP	7	44	0.125	21.7	2.17	5	278.5	128.3	6.4	25.7	4	self	8.75
IMP	7	45	0	24.8	2.48	16	844.4	340.9	6.6	21.3	3.2	out	8.75
IMP	7	49	0	26.5	2.65	9	577.4	218.2	4.4	24.2	5.5	out	8.75
IMP	7	50	0.25	23.3	2.33	6	421.9	180.8	6.8	30.1	4.4	self	8.75
IMP	8	1	0	8.9	0.89	12	252.2	284.4	6.2	23.7	3.8	out	8.13
IMP	8	2	0	27.2	2.72	6	175.8	63	5	10.5	2.1	out	8.13
IMP	8	4	0	27	2.7	5	116.1	42.9	8.4	8.6	1	out	8.13
IMP	8	5	0	14.2	1.42	8	184	129.3	7	16.2	2.3	out	8.13
IMP	8	7	0	15.6	1.56	15	361.1	231.5	7.4	15.4	2.1	out	8.13
IMP	8	10	0.125	33.2	3.32	17	994.8	299.9	11.6	17.6	1.5	self	8.13
IMP	8	11	0.125	12.6	1.26	8	188.6	150.1	5.2	18.8	3.6	self	8.13
IMP	8	12	0.125	27.3	2.73	9	233.5	85.5	7.4	9.5	1.3	self	8.13
IMP	8	20	0	27.7	2.77	9	102.5	39	3.4	4.3	1.3	out	8.13
IMP	8	27	0.125	32.6	3.26	16	272.8	83.8	4.6	5.2	1.1	self	8.13
IMP	8	28	0	16.8	1.68	15	374.3	222.4	6.6	14.8	2.2	out	8.13
IMP	8	29	0.25	25.4	2.54	21	917.8	361.3	9	17.2	1.9	self	8.13
IMP	8	30	0	16.5	1.65	15	667.6	404.6	8	27	3.4	out	8.13
IMP	8	36	0	26.2	2.62	14	919.2	350.4	10.6	25	2.4	out	8.13

IMP	8	40	0.125	17	1.7	10	277.4	163.5	6.2	16.3	2.6	self	8.13
IMP	8	48	0	32.2	3.22	15	739.6	229.7	5.6	15.3	2.7	out	8.13
IMP	9	1	0.125	30.9	3.09	14	468.4	151.6	8.2	10.8	1.3	self	10
IMP	9	4	0.125	28.8	2.88	4	287.1	99.7	10	24.9	2.5	self	10
IMP	9	8	0.125	30.2	3.02	14	380.3	125.8	5.6	9	1.6	self	10
IMP	9	9	0	14.3	1.43	16	444.9	311.1	6.8	19.4	2.9	out	10
IMP	9	11	0.125	22.7	2.27	11	348.9	153.7	6.8	14	2.1	self	10
IMP	9	12	0.25	21.1	2.11	17	332.3	157.5	8.4	9.3	1.1	self	10
IMP	9	13	0.25	20.1	2.01	20	1063.9	528.4	11.4	26.4	2.3	self	10
IMP	9	16	0.125	24.1	2.41	6	119.3	49.4	3.6	8.2	2.3	self	10
IMP	9	20	0	21.5	2.15	28	600.8	279.9	6.4	10	1.6	out	10
IMP	9	21	0.125	12.7	1.27	12	251.1	197.2	6.6	16.4	2.5	self	10
IMP	9	22	0	22.3	2.23	10	883.7	396.3	7.8	39.6	5.1	out	10
IMP	9	26	0.125	14.2	1.42	28	1401.5	987	8.2	35.2	4.3	self	10
IMP	9	28	0	24.7	2.47	6	177.5	72	5	12	2.4	out	10
IMP	9	29	0	29.4	2.94	20	812	275.9	7.4	13.8	1.9	out	10
IMP	9	32	0	12.5	1.25	6	161.8	129.8	2.6	21.6	8.3	out	10
IMP	9	37	0	24.9	2.49	10	819.3	329.5	8.6	32.9	3.8	out	10
IMP	9	38	0.375	13.8	1.38	26	881.7	638.9	10.8	24.6	2.3	self	10
IMP	9	43	0	20.6	2.06	7	370	179.6	8	25.7	3.2	out	10
IMP	9	44	0	21.6	2.16	4	169.3	78.4	8.5	19.6	2.3	out	10
IMP	10	26	0	23.1	2.31	26	1308.5	566.5	7.2	21.8	3	out	14.69
IMP	10	33	0	25	2.5	64	3865.4	1548.2	6.4	24.2	3.8	out	14.69
IMP	10	34	0.125	25.2	2.52	15	1833.2	728.4	6	48.6	8.1	self	14.69
IMP	10	35	0	26.4	2.64	25	2614.9	990.5	8.6	39.6	4.6	out	14.69
IMP	10	36	0.125	24.8	2.48	32	2401.8	969.8	9.4	30.3	3.2	self	14.69
IMP	10	37	0.375	30	3	28	1785.5	595.2	4	21.3	5.3	self	14.69
IMP	10	38	0.125	27.5	2.75	15	1379.4	502.2	12.8	33.5	2.6	self	14.69
IMP	10	39	0.25	24.1	2.41	28	976.2	405.1	8	14.5	1.8	self	14.69
IMP	10	40	0.625	24.9	2.49	38	1985.4	797.3	6.6	21	3.2	self	14.69
IMP	10	41	0.125	23.4	2.34	18	499.2	213.6	5.8	11.9	2	self	14.69
IMP	10	42	0.125	25.2	2.52	20	698	277.4	5.2	13.9	2.7	self	14.69
IMP	10	43	0.5	24.4	2.44	54	3115.5	1278.6	7.4	23.7	3.2	self	14.69

IMP	10	44	0.625	24	2.4	12	717.6	299.4	5.4	25	4.6	self	14.69
IMP	10	45	0.5	24.9	2.49	15	1053.4	423.1	8.2	28.2	3.4	self	14.69
IMP	10	46	0.125	23.9	2.39	22	740.8	309.5	4.2	14.1	3.3	self	14.69
IMP	10	47	0	20	2	21	1196.9	599.4	4.8	28.5	5.9	out	14.69
IMP	10	48	0.25	22.1	2.21	28	758.6	343.8	3.6	12.3	3.4	self	14.69
IMP	10	49	0	25.6	2.56	37	3020.8	1178.5	8.8	31.9	3.6	out	14.69
IMP	10	50	0	23.8	2.38	24	1088.8	457.5	6	19.1	3.2	out	14.69
IMP	11	2	0.125	26.4	2.64	14	376.1	142.3	6.6	10.2	1.5	self	12.19
IMP	11	3	0.125	21.8	2.18	6	316.2	144.8	5.8	24.1	4.2	self	12.19
IMP	11	4	0	26.8	2.68	14	638.9	238.1	6.4	17	2.7	out	12.19
IMP	11	7	0.75	27.5	2.75	24	1030.1	374.6	7.2	15.6	2.2	self	12.19
IMP	11	11	0.125	22.1	2.21	6	145.6	66	4.2	11	2.6	self	12.19
IMP	11	12	0	24	2.4	10	428.1	178.1	6.6	17.8	2.7	out	12.19
IMP	11	16	0.25	25.7	2.57	10	73.6	28	3	2.8	0.9	self	12.19
IMP	11	17	0.375	20.1	2.01	16	320.3	159.1	7.6	9.9	1.3	self	12.19
IMP	11	18	0.125	26	2.6	10	384.8	148.2	5.6	14.8	2.6	self	12.19
IMP	11	19	0.125	25	2.5	15	660.4	263.8	9.4	17.6	1.9	self	12.19
IMP	11	20	0	23.6	2.36	18	918.1	389.6	7.6	21.6	2.8	out	12.19
IMP	11	31	0.125	24.2	2.42	19	851	351.7	6.6	18.5	2.8	self	12.19
IMP	11	33	0.125	20.6	2.06	23	925.2	449.1	7.8	19.5	2.5	self	12.19
IMP	11	34	0	23.3	2.33	8	313.5	134.5	6.4	16.8	2.6	out	12.19
IMP	11	36	0	26.6	2.66	20	990.8	372.5	9.6	18.6	1.9	out	12.19
IMP	11	39	0	30.7	3.07	29	510.8	166.6	7	5.7	0.8	out	12.19
IMP	11	42	0.125	28	2.8	3	258.8	92.5	8	30.8	3.9	self	12.19
IMP	11	43	0	21.4	2.14	19	583.2	272.5	6.6	14.3	2.2	out	12.19
IMP	11	46	0.125	20.4	2.04	22	865.1	423.4	6.8	19.2	2.8	self	12.19
IMP	11	47	0	28.3	2.83	11	517.8	183.2	6	16.7	2.8	out	12.19
IMP	12	1	0	24.2	2.42	8	291.9	120.8	9.6	15.1	1.6	out	12.54
IMP	12	5	0.125	12.7	1.27	22	147.2	116.2	7.6	5.3	0.7	self	12.54
IMP	12	7	0	10.5	1.05	16	189.3	180.9	5.4	11.3	2.1	out	12.54
IMP	12	10	0	10.3	1.03	10	143.3	138.7	8.6	13.9	1.6	out	12.54
IMP	12	13	0.25	20.9	2.09	17	406.9	195	8.8	11.5	1.3	self	12.54
IMP	12	21	0.125	18.2	1.82	21	149	81.9	6.6	3.9	0.6	self	12.54

IMP	12	23	0	25.8	2.58	6	252.4	97.7	6	16.3	2.7	out	12.54
IMP	12	24	0	30.8	3.08	16	745.5	241.8	7	15.1	2.2	out	12.54
IMP	12	28	0.125	12	1.2	13	124.8	104.3	9.4	8	0.9	self	12.54
IMP	12	30	0.125	30.4	3.04	10	104.9	50	7	5	0.7	self	12.54
IMP	12	32	0	13.5	1.35	11	134.5	99.9	3.6	9.1	2.5	out	12.54
IMP	12	35	0	25	2.5	21	638.8	255.5	9.6	12.2	1.3	out	12.54
IMP	12	36	0.25	18.1	1.81	27	742.8	411.1	6.8	15.2	2.2	self	12.54
IMP	12	42	0	29.2	2.92	9	489.8	167.9	8.8	18.7	2.1	out	12.54
IMP	12	47	0.125	27.1	2.71	12	491.5	181.6	6.2	15.1	2.4	self	12.54

Variables in the Supplementary Data

The regions are SJV for San Joaquin Valley in the Central Valley of California and IMP for the Imperial Valley of California.

Field is the Field number within a region

Pl Self R is a plant or stem selfing rate, based on 8 seeds per stem

MW 10Seeds is the Average weight of 10 seeds

MSW is the average weight of one seed

Rac is the number of racemes per stem

TOT SW is the total seed weight (all seeds)

TOTSEED is the total number of seeds

Pods/Rac is the number of pods per raceme

Seeds/Rac is the number of seeds per raceme

Seeds/Pod is the number of seeds per pod

Sef.out is whether a stem was selfed or outcrossed

Field SR is the field selfing rate
